# High-affinity phosphate capture on MgO/Mg(OH)_2_-decorated wattle bark biochar: adsorption mechanism and regeneration performance

**DOI:** 10.1039/d6ra05212f

**Published:** 2026-10-12

**Authors:** Elham Jalali, Elizabeth Erasmus, Shahab Maghsoudi, Marietjie Schutte-Smith, Hendrik. G. Visser

**Affiliations:** a Chemistry Department, University of the Free State Bloemfontein 9300 South Africa Jalali.E@ufs.ac.za visserhg@ufs.ac.za; b Department of Chemistry, Faculty of Science, Shahid Bahonar University of Kerman Kerman Iran

## Abstract

MgO modified wattle bark biochar (MgO-BC) was produced by impregnating wattle bark with MgCl_2_ followed by pyrolysis at 600 °C and tested for the removal of phosphate from water. Material characterization showed the successful incorporation of MgO/Mg(OH)_2_ phases onto the biochar matrix and the presence of surface functionalities that are relevant for phosphate binding. Response surface methodology showed that solution pH, MgO-BC dosage, contact time, and initial phosphate concentration significantly influenced phosphate removal. The optimized conditions were pH 8.0, an MgO-BC dosage of 0.059 g in 10 mL solution, a contact time of 21 h, and an initial phosphate concentration of 64.6 mg P per L. Under these conditions, MgO-BC achieved approximately 99.5% phosphate removal. Equilibrium data over an extended initial phosphate concentration range (15–500 mg P per L) were best described by the nonlinear Langmuir isotherm (*q*_max_ = 11.42 ± 0.11 mg P g^−1^, *K*_L_ = 22.43 ± 1.58 L mg^−1^, *R*^2^ = 0.985), confirming high-affinity, monolayer uptake on a finite number of Mg-rich sites, whereas the nonlinear Freundlich and Temkin models provided inferior fits (*R*^2^ = 0.729 and 0.795, respectively). These model fits, combined with spectroscopic and XRD evidence for Mg–phosphate interactions, are consistent with a mechanism dominated by high-affinity surface reactions rather than weak physisorption. XRD patterns before and after adsorption confirmed that the main reflections of MgO/Mg(OH)_2_ did not shift after adsorption, indicating that the immobilization of phosphate was mainly due to inner-sphere complexation and poorly crystalline surface precipitation and not to bulk phase transformation. The initial phosphate removal efficiency of MgO-BC was found to be over 80% after five adsorption–desorption cycles, which confirms good regeneration potential. Based on these results, MgO-BC could be identified as an effective and reusable phosphate removal and recovery sorbent from aqueous systems.

## Abstract 2

MgO modified wattle bark biochar (MgO-BC) was produced by impregnating wattle bark with MgCl_2_ followed by pyrolysis at 600 °C and tested for the removal of phosphate from water.

## Graphical abstract

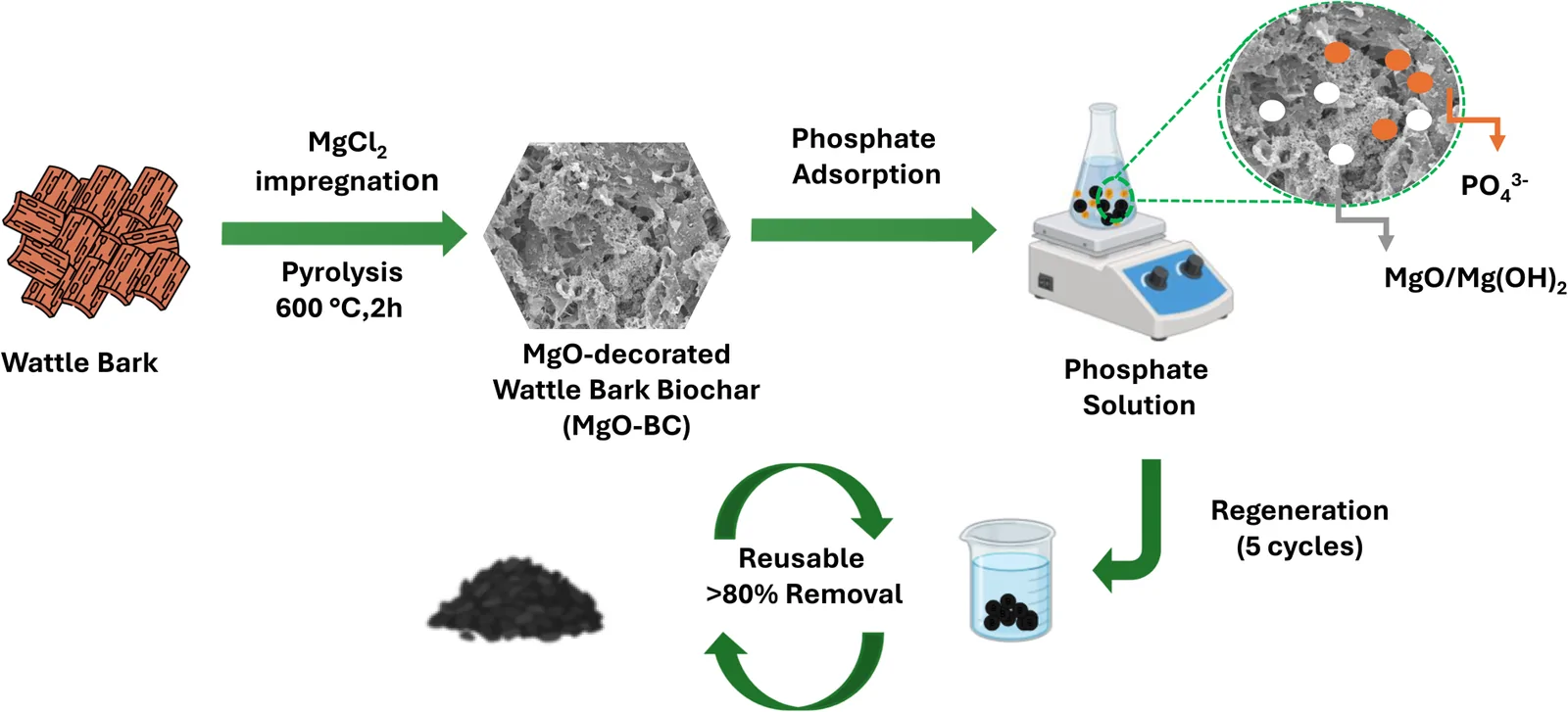

## 1. Introduction

An essential nutrient for plants, phosphorus (P) is a non-renewable resource.^1^ For crop growth in agricultural production, a significant amount of P fertilizer must be used.^2^ Large volumes of bio-liquid effluent were generated in tandem with the quick growth of large-scale livestock and poultry husbandry. As a result, recovering P from P-rich water bodies, including bio-liquid wastewater, is a crucial step in resolving the P resource issue. Phosphorus pollution, which results from the discharge of phosphorus into huge bodies of water including rivers, lakes, and the ocean, has grown to be a major global environmental problem.^3,4^ Urban sewage, livestock husbandry, and industrial operations are the main sources of this kind of pollution.^5^ Phosphorus and phosphate rock have recently been designated as Critical Raw Materials by the European Union under Regulation (EU) 2024/1252, reflecting their finite reserves, geographically concentrated supply, and absence of viable substitutes in food and feed production.^6^ World reserves of mineable phosphate rock are projected to be depleted within 50–300 years depending on consumption rates, while more than 90% of phosphorus used in agriculture is currently lost without recovery.^4^ The recovery of phosphate from P-rich wastewater therefore represents not only an environmental imperative but also a strategic contribution to closing the phosphorus cycle and reducing dependence on primary mineral resources.^7^ Eutrophication and environmental imbalances results from an excessive influx of phosphorus into the biogeochemical cycle. Ultimately, this nutrient overload triggers blooms and creates hypoxic ‘dead zones’, leading to a catastrophic loss of biodiversity within aquatic ecosystems. Thus, it is crucial to find efficient techniques for removing phosphorus from water. Adsorption has drawn a lot of interest recently as a promising method for removing phosphate from aquatic environments because of its high efficiency, low cost, and straightforward operation.^8^ The primary driver of nutrient loading and the primary cause of eutrophication is runoff water that containing high concentrations of nitrogen and phosphorus, frequently in the form of ammonium and phosphate.^9^ Adsorption,^10,11^ chemical treatment,^12^ and biological treatment^13^ are some of the methods that can be used to eliminate these nutrients.

The biological approach is relatively successful, its efficiency is limited by long hydraulic retention time to eliminate ammonium and phosphate and its performance is highly sensitive to operational fluctuations, such as temperature, pH, and organic loading rates. Chemical treatment, on the other hand involves high usage of chemicals, which makes the corresponding treatment expensive to operate and can also lead to secondary pollution. Adsorption is a method of choice due to its outstanding adsorption capability, convenience, and cost-effectiveness. It is normally applied in the last phase of the water/waste water treatment process to extract low-level contaminants in water and extract valuable adsorbates (organic solvents and precious metals).^14^

The inherent porosity of biochar facilitates the removal of many pollutants. Beyond its structural advantages, the integration of BC in phosphorus removal is not only favorable in order to reduce the costs involved, but also offers another green economic solution to resolve the phosphorus pollution issue in the process. However, it has limited or no adsorption capacity of phosphate because most of them contain biochar negative charges (OH^−^ and COO^−^) on the surface. Beyond the surface-charge limitation, pristine biochars produced from lignocellulosic biomass at 550–650 °C typically exhibit Langmuir monolayer capacities below 5 mg P g^−1^, which is insufficient for the treatment of phosphate-laden effluents at the concentrations commonly found in livestock wastewater (20–200 mg P per L).^15^ The valorisation of industrial by-product biomass, such as the spent bark of *Acacia mearnsii* (black wattle), a plantation tree cultivated at large scale in southern Africa for tannin extraction, as a biochar precursor simultaneously addresses waste management challenges and provides a low-cost, renewable sorbent matrix.^16^ To increase phosphate uptake, researchers have increasingly modified biochar with active substances like Mg and Al. While phosphate removal in these cases is primarily governed by ligand exchange and inner-sphere complexation, the risk of metals leaching poses a challenge. This potential for secondary pollution can decrease the overall viability of using metal-doped adsorbents in sensitive water bodies. Phosphate ions exhibit a high affinity for magnesium-based materials, particularly MgO, due to the presence of strong electrostatic interactions and the formation of magnesium-phosphate complexes. Consequently, incorporating MgO into biochar would significantly enhance its phosphate adsorption capacity of the biochar by providing active adsorption sites and increasing surface reactivity. Despite the considerable body of work on Mg-modified biochars, the use of *Acacia mearnsii* (black wattle) bark, an abundant industrial by-product from tannin extraction in South Africa, as a low-cost biochar precursor for MgO functionalization has not been reported. Furthermore, the systematic comparison of physical biochar pre-treatment strategies (freeze-drying, post-pyrolysis milling) with chemical MgO modification, combined with rigorous multi-factor optimization and mechanistic elucidation by converging isotherm, kinetic, XPS, and XRD evidence, has received limited attention in the literature. Unlike previously reported Mg-modified biochars, which typically prioritize maximizing Langmuir capacity through high Mg loadings or engineered nanostructuring,^17^ the present work demonstrates that a low-cost, previously unreported industrial by-product (spent *Acacia mearnsii* bark) can be converted into a phosphate sorbent combining a very high Langmuir affinity constant (*K*_L_) and near-zero separation factor (*R*_L_), properties of particular relevance for polishing low-residual-phosphate effluents, with demonstrated multi-cycle regeneration, while systematically isolating the contribution of surface chemistry from physical pre-treatment effects. This study therefore addresses these gaps by: (i) synthesizing and characterizing a series of wattle bark biochars with different physical and chemical modifications to isolate the individual contributions of textural and surface-chemical factors to phosphate uptake; (ii) applying a four-factor, five-level Central Composite Design (CCD) within Response Surface Methodology (RSM) to determine interaction effects among pH, MgO-BC dosage, contact time, and initial phosphate concentration, and to identify optimal operating conditions for maximum phosphate removal; (iii) establishing the adsorption mechanism through non-linear isotherm and kinetic modelling supported by XPS and powder XRD evidence before and after phosphate loading; and (iv) evaluating the regeneration potential and phosphate recovery over five consecutive adsorption–desorption cycles, with a view to positioning MgO-BC as a circular-economy-compatible sorbent. Batch experiments using synthetic phosphate solutions were employed to isolate and systematically quantify the effects of key process variables under controlled conditions.

## 2. Material and methods

### 2.1. Materials

The chemical reagents included analytical grade monopotassium phosphate (KH_2_PO_4_), magnesium chloride hexahydrate (MgCl_2_·6H_2_O, ≥98% purity), potassium dihydrogen phosphate (KH_2_PO_4_, ≥99% purity), and sodium hydroxide (NaOH, ≥99%) from Merck. All the experimental procedures of synthesis, washing, and adsorption were all conducted using deionized water. Stock solutions of phosphate were prepared by dissolving KH_2_PO_4_ in deionized water and kept for no more than 24 h. Wattle tree (*Acacia mearnsii*) bark was provided by NTE (Pietermaritzburg, South Africa), and dried at a temperature of 40 °C for 48 hours to dry the surface moisture content. For all the synthesis routes, the dried bark was ground in a planetary ball mill (Retsch MM 400, Retsch GmbH, Germany, 30 Hz, 10 min) and the ground product was further sieved with a 50 mesh sieve (0.25 mm). This particle size was chosen since it would be sufficient to have enough exposure of the surface during chemical activation and also to be consistent with known biomass pre-treatment regimens of biochar-based nanocomposite production. The powdered bark obtained after ball milling was kept in polyethylene bags to keep it dry at an ambient temperature.

### 2.2. Synthesis of pristine and physically modified biochar (WB-BC, FD-BC, and PT-BC)

Wattle bark was used to prepare three biochar variants to systemically isolate the individual and sequential effects of freeze-drying and secondary mechanical milling on their textural and surface properties. The materials were designated as Wattle Bark Biochar (WB-BC), Freeze-Dried Precursor Biochar (FD-BC) and Post-Treated Biochar (PT-BC), based on the sequence of physical treatments applied relative to the pyrolysis process. Table 1 presents a complete overview of all the synthesis conditions and the associated sample nomenclature. The three biochar variants were synthesized without any chemical activation agents to isolate the effects of the physical processing on biochar properties.

**Table 1:** Textural properties of wattle-bark-derived biochar variants determined from N_2_ adsorption at 77 K

Sample	*S*_BET_ (m^2^ g^−1^)	*S*_ext_ (*t*-plot, m^2^ g^−1^)	*V*_micro_ (cm^3^ g^−1^)	*V*_BJH,ads_ (cm^3^ g^−1^)
WB-BC	41.54	17.62	0.011433	0.05154
FD-BC	44.46	11.91	0.015966	0.04775
PT-BC	64.49	12.55	0.025965	0.05171
MgO-BC	48.98	26.70	0.010565	0.11325

### 2.3. Wattle bark biochar (WB-BC)-unmodified

WB-BC was made by direct pyrolysis of ball-milled wattle bark, with no chemical or other physical pre-treatment of the material, other than grinding and sieving. The pyrolysis experiment was carried out at 600 °C during a period of 2 hours in a continuous flow of nitrogen at a constant heating rate of 10 °C min^−1^. After pyrolysis, the furnace was cooled to room temperature with a constant flow of N_2_ to prevent oxidation of the hot carbon surface. The sample was then removed to avoid oxidation of the hot carbon surface. The resulting biochar was repeatedly washed with deionized water until the pH was stable. The solid was dried in the oven at 80 °C.

### 2.4. Freeze-dried precursor biochar (FD-BC)-pre-pyrolysis freeze-drying

To determine the influence of freeze-drying the lignocellulosic precursor before pyrolysis on the pore structure and surface area of the resultant biochar, FD-BC was synthesized. The ball-milled wattle bark was added in a 1 : 10 (w/v) ratio of solid : liquid in deionized water and mixed using magnetic stirring for a period of 1 h at room temperature to achieve a high degree of wettability within the biomass matrix. The suspension was frozen in pre-weighed containers and kept frozen at −18 °C for 12 h to create a uniform ice matrix, followed by freeze-dried under vacuum (pressure 10 Pa or lower) for 24 h until a steady dry mass was obtained.

This freeze-drying step was added based on the assumption that the ice sublimation within the hydrated biomass matrix preserves the expanded, water-swollen, pore architecture. By substituting liquid water with a sublimable solid phase, this approach avoids the capillary-driven pore collapse that occurs with traditional oven drying. This ice-templating process has been noted to produce hierarchical pore networks in biomass-derived carbons which cannot be obtained through direct drying. The freeze-dried precursor was pyrolyzed in the same conditions as WB-BC (600 °C 2 h N_2_ 10 °C min^−1^) followed by the same washing and drying procedure was performed. This product was named FD-BC.

### 2.5. Post-treated biochar (PT-BC)-post-pyrolysis sequential physical treatment

The production of PT-BC was conducted to determine whether sequential post-pyrolysis freeze-drying and secondary ball milling could be used to break up aggregated microstructures in a pre-formed biochar and expose more inner surface area, without changing the overall chemical composition produced during pyrolysis. This method is fundamentally different to FD-BC, in that the physical treatment is performed after, not preceding pyrolysis, thus separating out the impact of carbon matrix formation and post-formation textural modification.

WB-BC (according to previous section) was re-dispersed in deionized water (1 : 10 w/v) and stirred. The suspension was allowed to freeze and freeze dried in a vacuum (pressure less than 10 Pa) for 12 and 24, h respectively. The freeze-dried sample was then subjected to secondary ball milling under the same conditions as in previous section. The resulting solid was transferred to a desiccator before characterization. This product was named PT-BC. Admittedly, the structural responsiveness of a pre-existing, inflexible carbon matrix to freeze-drying can be less than that of a fresh lignocellulosic precursor, as the condensation of the carbon structure is irreversible during pyrolysis.

### 2.6. Synthesis of MgO-modified wattle bark biochar (MgO-BC)

The active sites based on magnesium were introduced to phosphate adsorption by pyrolyzing a MgCl_2_ impregnated wattle bark precursor. Briefly, 20 g of ball-milled and sieved wattle bark was dispersed in, 250 mL aqueous magnesium chloride hexahydrate (MgCl_2_·6H_2_O, 1.0 mol L^−1^). The suspension was ultrasonicated for 1 h at room temperature to promote penetration of Mg^2+^ ions in the biomass matrix and uniform impregnation of the lignocellulosic structure. The solution was magnetically stirred until the solution reached equilibrium between Mg^2+^ and surface functional groups on the bark, after 24 h at ambient temperature. Filtration was used to collect impregnated solids, followed by drying in an oven at 60 °C overnight to remove any remaining moisture content, and gently disaggregated with a spatula.

The dried MgCl_2_-impregnated precursor was pyrolyzed in a tubular furnace at 600 °C for 2 h under a continuous flow of high-purity argon at a heating rate of 10 °C min^−1^. After pyrolysis, the furnace was cooled to room temperature under continuous argon flow to prevent oxidation of the hot material. The resulting MgO-modified biochar (MgO-BC) was washed repeatedly with deionized water (to remove loosely bound inorganic salts and soluble impurities), and dried at 80 °C. The MgO-BC, which was stored in a desiccator.

### 2.7. Characterization

Fourier-transform infrared spectroscopy (FTIR) analysis of the functional groups was conducted using a Tensor 27 spectrometer equipped with Pike miracle single bounce diamond ATR crystal and analyzed with OPUS v1.1 software. Data was refined with microactive v1.01 software. In the case of thermo-gravimetric analyses (TGA), it was carried out using a nitrogen atmosphere in the Mettler-Toledo TGA/SDTA851. STAR SW 8.10 software was used to analyze the TGA thermograms. The surface area and porosity were determined on a Micromeritics ASAP 2020 surface area and porosity analyzer and ASAP v2.0 programs. A JSM-7800 F-Extreme-resolution analytical field emission instrument was utilized to obtain field emission scanning microscope (FESEM), and energy dispersive X-ray spectroscopy (EDS). An Agilent Cary 60 UV-vis spectrometer was used to collect the UV-visible spectra. Powder X-ray diffraction (XRD) patterns were recorded on a Bruker D8 Advance diffractometer using Cu Kα radiation (*λ* = 1.5406 Å) at 40 kV and 40 mA. Data were collected in coupled 2*θ*/*θ* geometry from 5–90° 2*θ* with a step size of 0.020°, and evaluated using DIFFRAC.EVA software. Phase identification was performed by comparison with the ICDD PDF database.

### 2.8. Adsorption experiments

The performance of WB-BC, FD-BC, PT-BC and MgO-BC in removing phosphate was determined by batch adsorption experiments. Usually, 10 mL of phosphate solutions at the appropriate concentration were combined with a specific quantity of adsorbent. After that, the suspension was agitated for a predetermine time. Finally, the suspension was filtered through 0.45 µm membrane filters, and the phosphate concentration was measured at 880 nm using the ascorbic acid–molybdenum blue method (Standard Methods 4500-P D). The removal effectiveness *R* (%) and adsorption capacity (*q*, mg g^−1^) of MgO-biochar towards phosphate were calculated using eqn (1) and (2), respectively:1
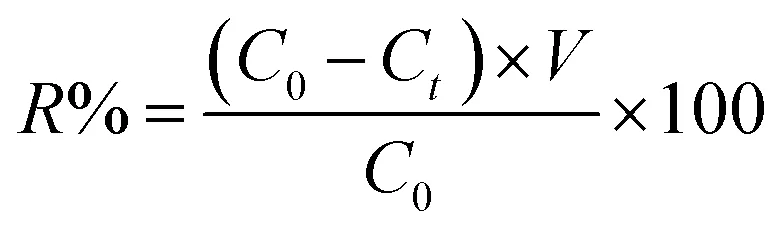
2
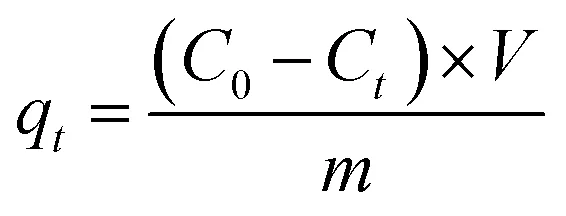
*V* is the volume of phosphate solution (L), *m* is the mass of biochar utilized (g), *C*_0_ is the starting phosphate concentration (mg^−1^), and *C*_*t*_ is the phosphate concentration (mg^−1^) at a specific time. At equilibrium, *C*_*t*_ and *q*_*t*_ are denoted as C_e_ and *q*_e_, respectively.

Equilibrium isotherm experiments were conducted at initial phosphate concentrations (*C*_0_) ranging from 15 to 500 mg P per L under the optimized conditions (pH 8.0, 0.059 g MgO-BC in 10 mL solution, contact time 21 h). All reported *q*_e_ values were verified against mass-balance calculations using eqn (2).

#### 2.8.1. Effect of ionic strength

To assess the role of background electrolyte on phosphate uptake, batch adsorption experiments were conducted under the optimized conditions established by the CCD pH 8.0, 0.059 g MgO-BC in 10 mL, initial phosphate concentration *C*_0_ = 64.61 mg P per L, in the presence of NaNO_3_ as the background electrolyte at concentrations of 0 (control), 0.001, 0.01, 0.1 and 0.5 mol L^−1^. All experiments were performed in triplicate and results are reported as mean ± standard deviation.

### 2.9. Experimental design and CCD optimization

Response surface methodology (RSM) was used to systematically examine the effects of significant operational variables on phosphate adsorption by MgO-biochar using a four-factor, five-level central composite design (CCD) in Design-Expert software (Stat-Ease Inc., USA). Initial solution pH (*X*_1_), MgO-biochar dosage (*X*_2_, g), contact time (*X*_3_, min) and initial phosphate concentration (*X*_4_, mg L^−1^) were chosen as independent variables, since they are generally considered the most significant factors determining the adsorption performance of a batch system. The coded and actual levels of each experimental factor were selected based on single-factor screening trials conducted prior to the CCD, in which each variable was varied independently while all others were held at a central value, and on a review of the relevant literature on Mg-modified biochar phosphate sorbents. These preliminary trials confirmed that the pH range 3.5–11.0, dosage range 0.001–0.100 g, contact-time range 8–24 h, and concentration range 5–100 mg P per L bracketed the region of significant variation in removal efficiency.

A rotatable CCD was generated with 30 runs (16 factorial, 8 axial and 6 centre points) to allow the determination of pure error and to test for model curvature. Five levels (−2, −1, 0, +1, +2) of each factor were investigated in terms of the chosen minimum, low, central, high and maximum values set in the software, respectively. The batch adsorption experiments were carried out according to the design matrix and the response (*Y*) was the percent phosphate removal (*R*) based on the difference between initial and final phosphate concentrations. The response data were fitted to a second-order polynomial:
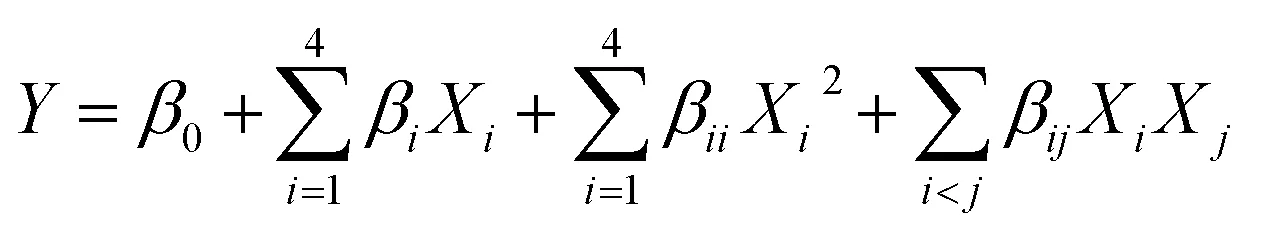
where *β*_0_ is the intercept, *β*_*i*_ are the linear coefficients, *β*_*ii*_ are the quadratic coefficients and *β*_*ij*_ are the interaction coefficients. To determine the statistical significance of the model and individual terms, analysis of variance (ANOVA) was used, based on *F*- and *p*-values, coefficients of determination (*R*^2^ and adjusted *R*^2^), and lack-of-fit tests, as is common in RSM analysis of adsorption experiments. The suitability of the fitted model was also assessed by comparing predicted and experimental responses and reviewing residual plots to ensure normality, constant variance and absence of systematic patterns. The desirability-function approach in Design-Expert was then used to numerically optimize the factors within the experimental ranges while maximizing phosphate removal. The software derived an overall desirability and suggested the optimal combinations of pH, MgO-biochar dosage, contact time and initial phosphate concentration. To confirm the reliability of the optimization, additional experiments were conducted at the predicted optimum conditions, and the experimentally determined phosphate removal efficiencies were compared with the model-predicted values.

### 2.10. Adsorbent regeneration and phosphate recovery

Phosphate-loaded MgO-BC, obtained under the optimized adsorption conditions (pH 8.0, 0.059 g adsorbent in 10 mL, 64.6 mg P per L, contact time 21 h), was separated by filtration (0.45 µm), rinsed with deionized water, and dried at 25 °C. The dried solid (0.059 g) was then contacted with 10 mL of 0.10 mol per L NaOH for 2 h at 25 °C under agitation to desorb bound phosphate. The regenerant was analyzed for phosphate by the same ascorbic acid–molybdenum blue method at 880 nm described in Section 2.8, and the desorption capacity was calculated by mass balance. The regenerated solid was rinsed four times with deionized water, dried, and reused for the next adsorption cycle. This procedure was repeated for five consecutive adsorption–desorption cycles.^18–20^

## 3. Results and discussion

### 3.1. Characterization of MgO-biochar

#### 3.1.1. FTIR characterization

The FTIR spectra of the unmodified wattle bark biochar (WB-BC) and the MgO-modified biochar (MgO-BC) were captured in the range 4000–400 cm^−1^ (Fig. 1a). The raw biochar has a broad band at 3400–3200 cm^−1^, which is assigned to OH stretching vibrations of hydroxyl groups present on the surface of the biochar and adsorbed water as is usually observed in biochar made from of lignocellulosic biomass.^21^ Weak absorptions around 2920–2850 cm^−1^ are due to the aliphatic C–H stretching of the remaining organic moieties. The C=O stretching vibrations of carbonyl and carboxylic groups related to conjugated aromatic structures are allocated to the band in the range 1700–1600 cm^−1^. The intricate envelope of between 1450 to 1000 cm^−1^ is primarily C–O stretching and O–H bending vibrations of phenolic, alcoholic and carboxylate functionalities, suggesting the occurrence of oxygenated groups capable of engaging in surface complexation during the adsorption.

**Fig. 1:**
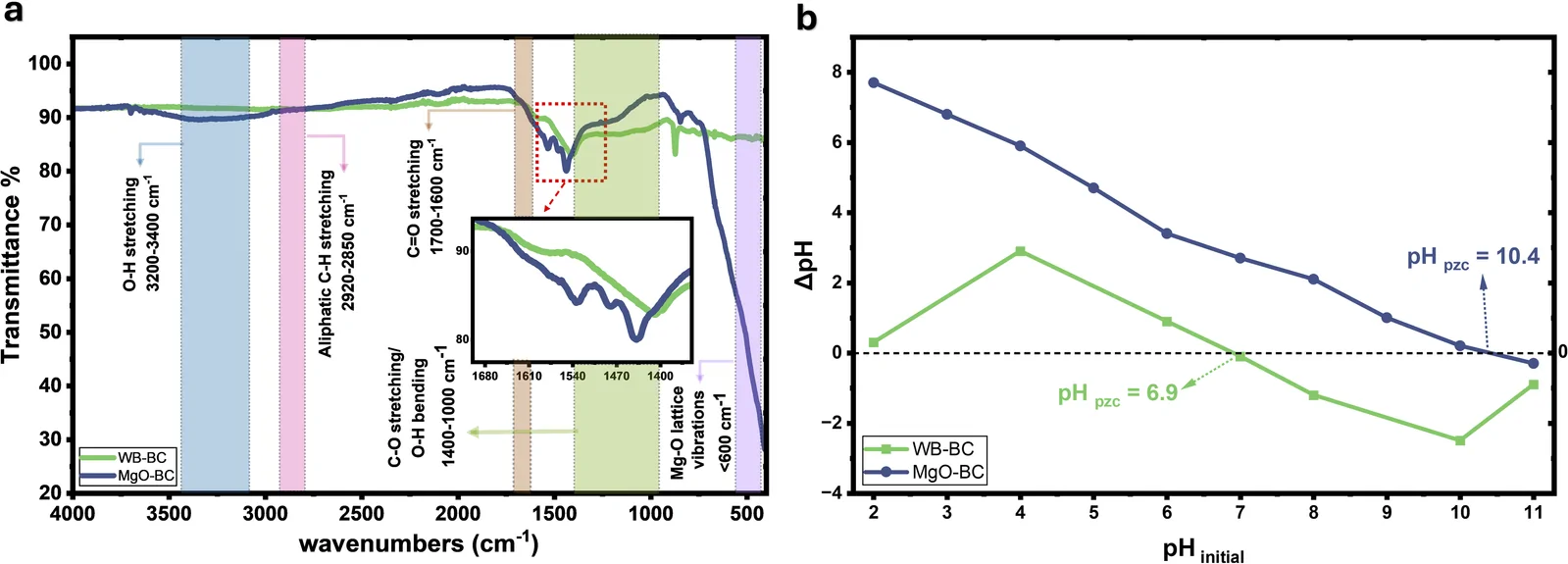
Surface chemistry and charge properties of WB-BC and MgO-BC: (a) FTIR spectra showing evolution of O–H, C–H, C=O and Mg–O vibrations after MgO loading; FTIR spectra were recorded in ATR mode at room temperature over 4000–400 cm^−1^. (b) pH-drift curves used to determine the point of zero charge (pH_PZC_ = 6.9 for WB-BC and 10.4 for MgO-BC). pH-drift measurements were conducted in 0.01 mol L^−1^ NaCl (0.05 g adsorbent in 25 mL) with 24 h equilibration at 25 °C.

The general spectral profile of the carbon matrix in MgO-BC after impregnation and pyrolysis remains broadly similar to that of WB-BC; however, several distinct changes confirm the incorporation of magnesium species. The O–H stretching region exhibits increased intensity and sharpening, consistent with an enrichment of surface hydroxyls, including Mg–OH groups, and enhanced hydration of the modified surface. Furthermore, the emergence of a distinct band below approximately 600 cm^−1^ is attributable to Mg–O lattice vibrations, while Mg–OH-related features have also been reported in MgO-modified biochars used for phosphate capture.^22–24^ Small changes in the 1450–1000 cm^−1^ region further suggest that Mg^2+^ interacted with pre-existing oxygen-containing functionalities on the biochar surface, thereby generating Mg–O/Mg–OH centres that can act as high-affinity sites for phosphate binding. The change in relative intensity near 1500 cm^−1^ in MgO-BC is attributed to the attenuation of aromatic C=C ring-stretching bands from the carbonaceous matrix, coinciding with the emergence of asymmetric O–C–O stretching contributions from Mg-carboxylate or Mg-carbonate surface interactions, consistent with reports on Mg-modified lignocellulosic biochars.

#### 3.1.2. pH drift and point of zero charge (pH_PZC_)

The point of zero charge (PZC) for both unmodified and MgO-modified biochar (MgO-BC) was determined using the pH-drift method in 0.01 mol L^−1^ NaCl background electrolyte (Fig. 1b). The change in pH (ΔpH = pH_f_ − pH_i_) was plotted against the initial pH (pH_i_), with the pH_PZC_ identified as the point where ΔpH = 0. This established method is a good predictor of the pH at which the biochar surface maintains a net neutral charge.^25^

In the case of the pristine wattle bark biochar, ΔpH was positive at low pH_i_ and gradually decreased with increasing initial pH, reaching zero in the pH_i_ range 6–7 (ΔpH = 0.9 and −0.1, respectively). As a result, the pH_PZC_ value for the pristine biochar is approximately 6.9, which is comparable with many lignocellulosic biochar produced at 550–700 °C. At pH < 7.0 the surface is positively charged and hence preferred for the sorption of anions, while at pH > 7.0 the surface becomes negatively charged and preferred for the sorption of cations. This relatively low pH_PZC_ and the low inorganic content contribute to explaining the low intrinsic selectivity of the pristine biochar towards phosphate anions.

In comparison, MgO-BC has a higher pH_PZC_. In the case of MgO-BC, the ΔpH value was strongly positive over the whole range of acidic and neutral pH_i_ and only approached zero between pH_i_ 10 and 11 (ΔpH = 0.2 and −0.3, respectively), resulting in a pH_PZC_ of approximately 10.4. The significant increase in pH_PZC_ following MgO addition is in agreement with the formation of basic MgO/Mg(OH)_2_ domains on the biochar surface and has been found in other Mg-modified biochar and Fe/Mg-biochar composites. Given the significantly lower pH range for phosphate adsorption used in this work, the MgO-BC surface is likely to be positively charged during the adsorption experiments, which could result in electrostatic interactions between protonated Mg–OH^+^ surface sites and anionic phosphate species (H_2_PO_4_^−^, HPO_4_^2−^).

Thus, the high pH_PZC_ and presence of a large amount of MgO/Mg(OH)_2_ sites on MgO-BC creates a strong electrostatic affinity towards phosphate adsorption, in addition to specific interactions, such as surface complexation and precipitation of magnesium phosphate phases. In contrast, the low pH_PZC_ and predominantly carbonaceous surface of the unmodified biochar limits its function to non-specific interactions, which is consistent with previous studies that show Mg modification significantly improves phosphate sorption relative to unmodified biochar.^23,26–28^

#### 3.1.3. Thermal stability and implications for adsorption

The thermal stability of MgO-BC was studied by thermogravimetric analysis (TGA) in an inert environment in the temperature range 30–750 °C (Fig. 2). The TG curve reveals high stability of the material with a total weight loss of only around 12–15 wt% over the whole temperature range, which is in agreement with the presence of a condensed aromatic carbon structure and the inorganic components (MgO/Mg(OH)_2_). Such low mass losses have also been observed for Mg-modified biochar for high-temperature use, as well as for phosphate sorbents containing MgO-biochar composites.^29^

**Fig. 2:**
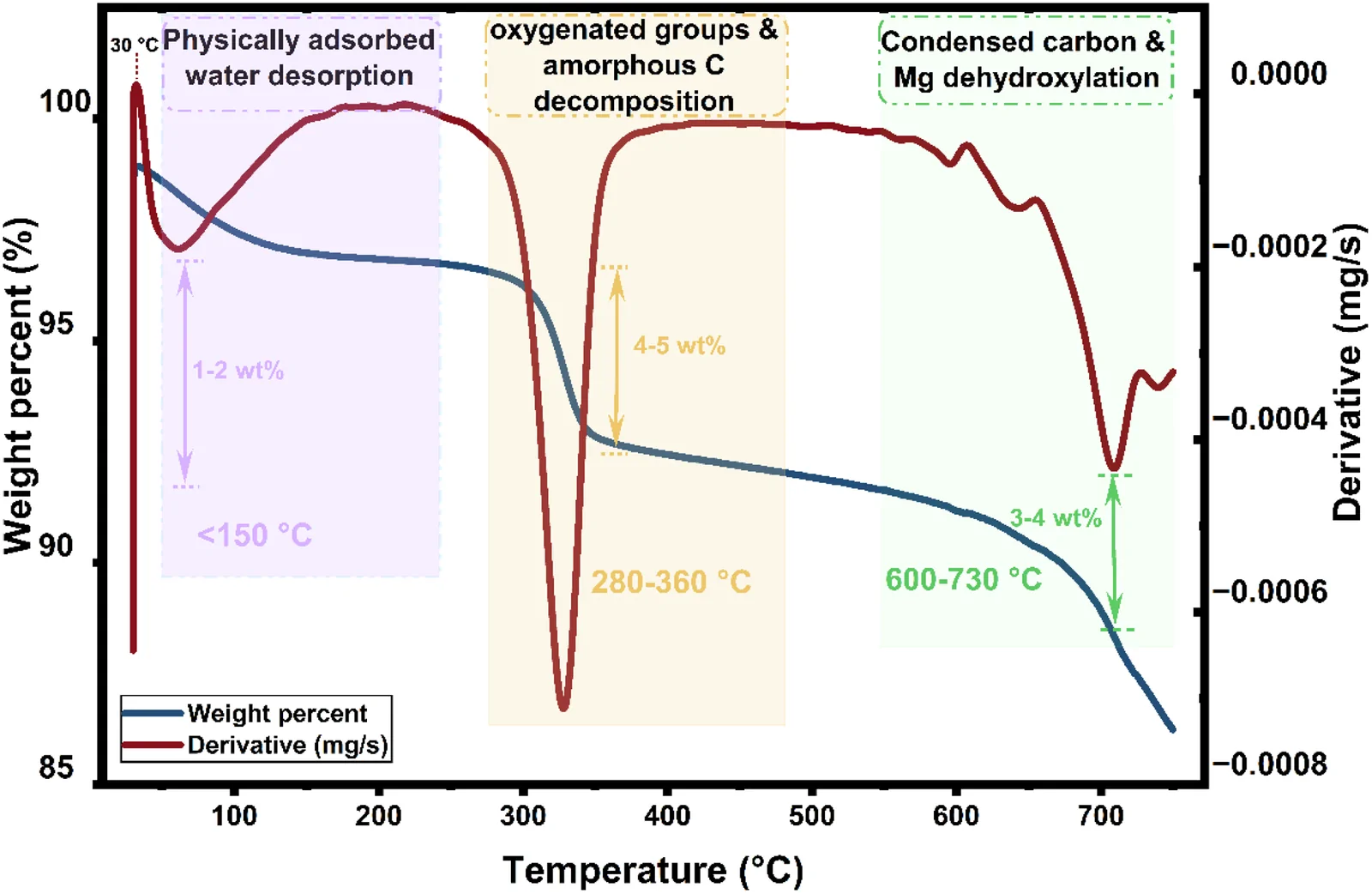
Thermogravimetric analysis (TGA) and derivative thermogravimetry (DTG) of MgO BC under N_2_ atmosphere in the range 30–750 °C.

The first, small mass-loss event below about 150 °C is related to the desorption of physically bound water and a small fraction of weakly bound species, as indicated by the small peak in the derivative thermogravimetric (DTG) curve. This behavior is characteristic for biochar and MgO-containing materials that contain hydrophilic surface hydroxyl groups that can adsorb water. The second and main DTG peak appears in the temperature range of approximately 280–360 °C where a larger mass loss (4–5 wt%) is observed due to the decomposition of residual labile oxygenated surface functional groups and less ordered aliphatic or amorphous carbon structures that did not decompose during pyrolysis at 600 °C.^30–32^

The thermal stability of MgO-BC has important consequences for both its adsorption efficiency and long-term viability. First, the presence of mainly inert aromatic carbon matrix and inorganic MgO ensures structural integrity at temperatures relevant to environmental applications and thermal regeneration (≤200–300 °C), which is beneficial for reusability. Second, the small mass loss near 200–300 °C for labile functional groups means that most of the Mg–O/Mg–OH active sites and strongly bound oxygenated functional groups are preserved. This suggests that phosphate uptake are governed by stable chemical processes, such as inner-sphere surface complexation and surface precipitation of magnesium phosphate compounds, rather than weak physisorption. Third, the remaining fraction of labile groups may help with initial wetting and activation of MgO-BC surface upon contact with water, which may enhance the surface wettability and allow phosphate ions to reach internal active sites, as has been reported for other Mg- or Fe/Mg-modified biochar.^33^

The TGA/DTG analyses confirms the structural and thermal stability of MgO-BC carbon-inorganic matrix. This, combined with the high pH_PZC_ and MgO content shown by pH-drift, is a consistent indication of a stable, reactive sorbent material. Such properties indicate that a chemisorption-dominated mechanism of phosphate removal can be maintained under a broad spectrum of environmental conditions.

#### 3.1.4. BET and *t*-plot results

Nitrogen adsorption–desorption isotherms of WB-BC, FD-BC, PT-BC and MgO-BC measured at 77 K display type IV, intermediate-pressure hysteresis loops, consistent with mesoporous slit-like structures of lignocellulosic biochar.^34^ BJH pore size distributions reveal wide mesopore distributions primarily in the 5–70 nm size domain, with MgO-BC exhibiting much larger differential pore volumes (calculated per unit pore size) in the 10–40 nm diameter range than the carbon-only biochar (Fig. 3a and b). These mesoporous structures are in good agreement with previous reports on wood-derived and Mg-doped biochar and facilitate fast intraparticle-ion diffusion from water. Textural parameters derived from BET and *t*-plot analyses are summarized in Table 1.

**Fig. 3:**
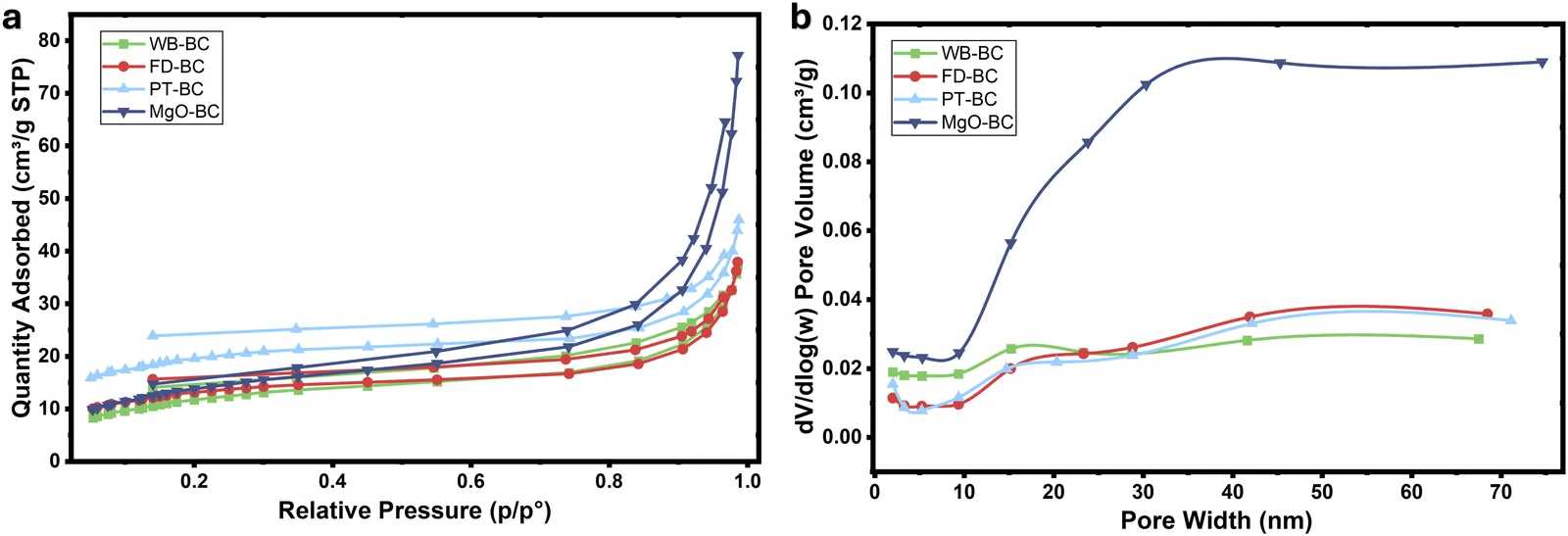
Textural properties of wattle bark-derived biochar determined from N_2_ adsorption at 77 K. (a) N_2_ adsorption–desorption isotherms of WB BC, FD BC, PT BC and MgO BC, showing type IV behavior with H type hysteresis indicative of mesoporous structures; (b) BJH pore size distributions calculated from the desorption branches. Fixed conditions: N_2_ adsorption–desorption measured at 77 K after degassing at 120 °C for 12 h under vacuum.

The pristine WB-BC shows a moderate BET surface area (*S*_BET_) of 41.54 m^2^ g^−1^ and low micropore volume (*V*_micro_) of 0.011433 cm^3^ g^−1^, as common for non-activated biochar produced at about 600 °C.^35,36^ The FD-BC shows a slightly higher *S*_BET_ (44.46 m^2^ g^−1^) but a larger *V*_micro_ (0.015966 cm^3^ g^−1^), so the preservation of the swollen precursor structure probably facilitates micropore development. However, the post-treated PT-BC exhibits a more significant increase in both *S*_BET_ (64.49 m^2^ g^−1^) and *V*_micro_ (0.025965 cm^3^ g^−1^) compared with the WB-BC, suggesting that the post-treatment protocol more efficiently opens internal domains of the carbon matrix.^35,37,38^

MgO-BC shows a moderate *S*_BET_ (48.98 m^2^ g^−1^) but the largest external surface area (26.70 m^2^ g^−1^) and a *V*_micro_ (0.010565 cm^3^ g^−1^) equal to or smaller than that of the carbon-only biochar. This suggests that the MgCl_2_ treatment and co-pyrolysis process produce extra accessible external and mesoporous surface with MgO/Mg(OH)_2_ decoration, rather than significantly enhancing micropores, as reported for MgO-modified biochar without harsh physical activation. Similar low to moderate *S*_BET_ (20–150 m^2^ g^−1^) but significantly enhanced external surface and mineral phase content have been reported for MgO-biochar and Mg/Fe-biochar composites used to remove phosphate.^39–41^

#### 3.1.5. Implications for adsorption behavior and mechanism

The pore structure development of the four materials has implications for phosphate adsorption. The modest micropore volumes of all these materials indicate that adsorption will be dominated by processes on external and mesopore surfaces rather than micropore filling. This aligns with prevailing opinion that biochar's adsorption efficiency is more closely related to the accessible surface area and surface chemistry than to the total micropore volume. While the increased *S*_BET_ and *V*_micro_ of PT-BC relative to WB-BC and FD-BC suggest that post-treatment increases the internal surface area, thereby potentially increasing non-specific interactions such as van der Waals, hydrogen bonding, but in the absence of MgO these interactions typically lead to limited phosphate capacities.^42,43^

In addition to the BET and *t*-plot data, the BJH adsorption cumulative pore volumes (Table 1) give a clearer picture of how mesoporous the different biochars are. WB-BC, FD-BC and PT-BC all have similar total BJH pore volumes of about 0.051–0.052 cm^3^ g^−1^, whereas MgO-BC shows a much larger value of 0.113 cm^3^ g^−1^, confirming the mesopore-rich structure suggested by the N_2_ isotherms and BJH curves. Combined with its higher external surface area, this greater mesopore volume means that MgO-BC contains a larger fraction of easily accessible pores in the 5–70 nm range, which promotes faster intraparticle diffusion of phosphate to Mg-rich surface sites and helps to explain its superior adsorption performance compared with the carbon-only biochars.

For MgO-BC, the increase in *S*_BET_ and large increase in *S*_ext_, combined with the mesopore-dominated BJH pore-size distribution, indicate that solution-phase phosphate can readily access Mg-rich surface domains, providing favorable conditions for specific chemical interactions at these sites. The detailed adsorption mechanism involving electrostatic attraction, inner-sphere complexation and surface precipitation at MgO/Mg(OH)_2_ domains is discussed in Sections 3.4 and 3.5 using equilibrium, kinetic and XRD evidence.

The BET and BJH results indicate that while physical treatments (FD-BC, PT-BC) increase the surface area and micropore volume of wattle bark biochar samples, MgO addition alters the structure and accessibility of active sites through an increase in external mesopore area and the formation of Mg-rich sites. This architecture has a logical explanation for the better phosphate sorption behavior of MgO-BC than the carbon-only biochar, and is consistent with reports of MgO-modified biochar as reusable phosphate sorbents.^22,44–47^

#### 3.1.6. FESEM

The micrographs of the unmodified wattle bark biochar and the magnesium oxide-modified biochar (MgO-BC) are displayed in Fig. 4a–f. The pristine biochar consists of relatively dense, plate-shaped particles with smooth, fractured surfaces and relatively few visible micro-pores at 1500× and 4000× magnification (Fig. 4a and b), whereas MgO-BC shows a much more fragmented, aggregate-like morphology at higher magnifications of 14 000× and 30 000× (Fig. 4d and e). These types of structures are common for lignocellulosic biochar pyrolyzed at 600 °C and generally offer only a small surface for anion adsorption. Accordingly, the EDS spectrum of the unmodified biochar (Fig. 4c) shows a predominance (90 wt%) of carbon, with only minor amounts of oxygen and traces of calcium. This suggests that the biochar is primarily composed of a carbon matrix with very limited amounts of inorganic elements that may serve as high-affinity sites for phosphate binding.

**Fig. 4:**
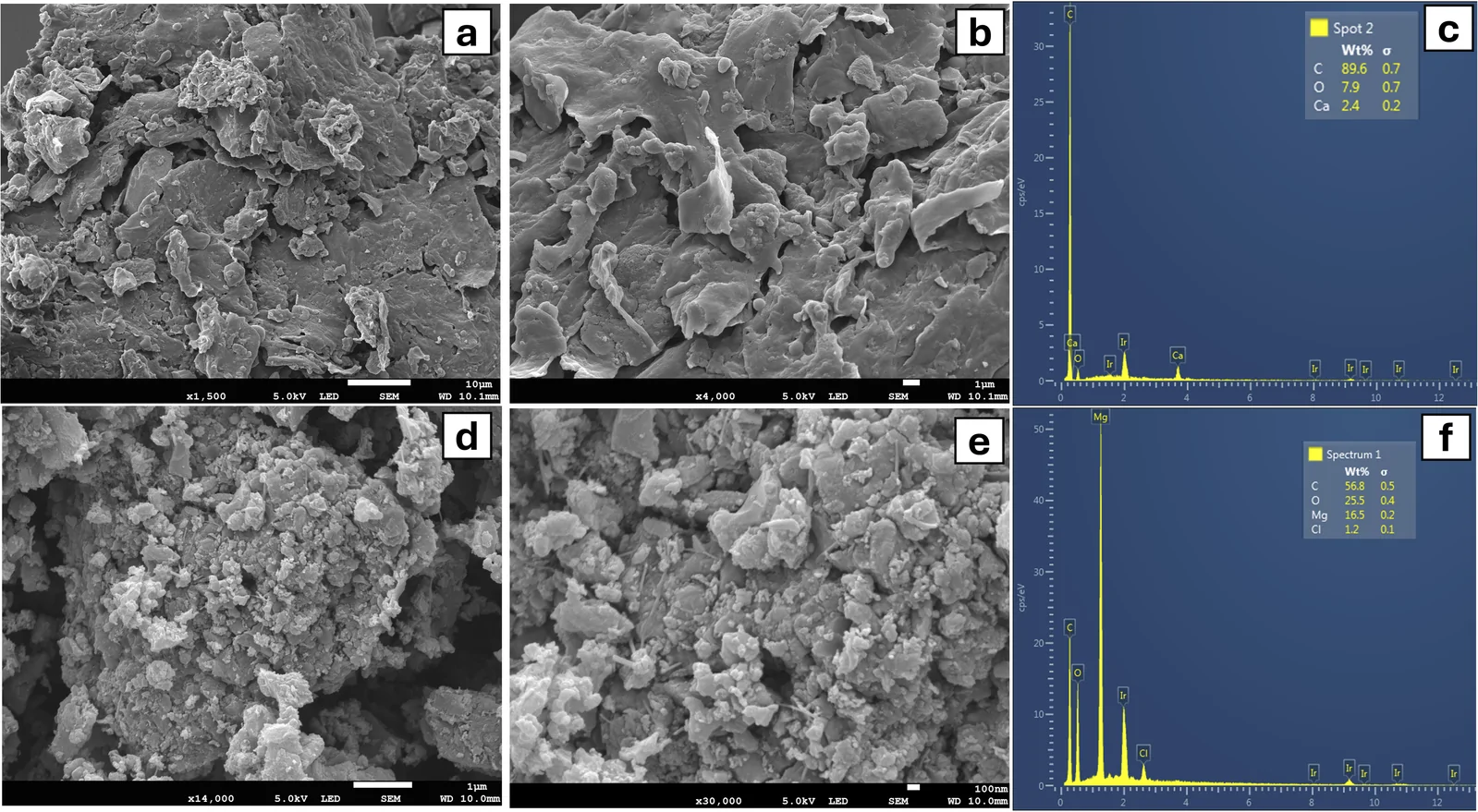
FESEM micrographs and EDS spectra of unmodified and MgO-modified wattle bark biochar. (a and b) WB-BC at 1500× and 4000× magnification, showing dense plate-like carbon particles with limited visible micro-porosity. (c) Corresponding EDS spectrum of WB-BC, dominated by carbon with minor oxygen and trace inorganic elements. (d and e) MgO-BC at 14 000× and 30 000× magnification, revealing a highly textured surface composed of fine aggregates coating the carbon matrix. (f) EDS spectrum of MgO-BC, confirming successful Mg loading with increased oxygen content and the presence of Mg and residual Cl associated with MgO/Mg(OH)_2_ domains. Scale bars: 10 µm (a and d), 1 µm (c), and 100 nm (d).

By comparison, the micrographs of the activated MgO-BC (Fig. 4d and e) reveal a much more eroded and fragmented surface, which consists of clusters of fine particles coating and bridging the carbon plates. This particle coating is in line with the *in situ* precipitation and deposition of MgO/Mg(OH)_2_ aggregates during the MgCl_2_ impregnation and pyrolysis processes. This fluffy and cluster-like MgO structure has been reported for MgO-modified rice straw and peanut shell biochar, which is associated with both an increased specific surface area and an increase in the number of accessible Mg active sites. The more porous and irregular structure of MgO-BC is likely to affect mass transfer and increase the number of accessible sites for phosphate binding, compared with the relatively flat pristine biochar.^48^

The EDS spectrum of MgO-BC (Fig. 4f) confirms the successful loading of Mg, the C content is significantly reduced to 57 wt%, O is now increased (25 wt%) and there is a strong Mg peak (16 wt%) that appears, along with a minor Cl contribution from MgCl_2_. This C–O–Mg composition is consistent with other Mg-modified biochar produced by impregnation or co-pyrolysis.^49,50^ In such biochar, the Mg content is usually in the 5–20 wt% range and is positively associated with enhanced phosphate adsorption. The presence of Mg and increased oxygen content suggests the formation of MgO/Mg(OH)_2_ phases dispersed on an oxygen-rich carbon matrix, which can interact with phosphate in multiple ways: (i) electrostatic attraction between protonated Mg–OH^+^ surface sites and anionic phosphate species (predominantly H_2_PO_4_^−^ and HPO_4_^2−^) when the solution pH is below the pH_PZC_ of MgO-BC (pH_PZC_ = 10.4), resulting in a positively charged adsorbent surface, (ii) inner-sphere complexation with H_2_PO_4_^−^ or HPO_4_^2−^ and MgO/MgOH groups, and (iii) surface precipitation of magnesium phosphate minerals like Mg_3_(PO_4_)_2_.^51–53^ Our results are in line with the broader literature on MgO-biochar systems, where SEM-EDS studies commonly show that unmodified biochars have few inorganic active sites and comparatively poor phosphate removal, whereas Mg-modified biochars possess abundant Mg-rich particles on their surfaces and markedly higher P uptake. For example, MgO-loaded agricultural biochars and related Mg-functionalized biochars have been reported with phosphate adsorption capacities from tens up to several hundred mg P g^−1^, depending on Mg loading, precursor and conditions, and in some cases exceeding 150–200 mg P g^−1^. In many of these studies, equilibrium data are well fitted by Langmuir isotherms and the kinetics by pseudo-second-order rate expressions, which is consistent with high-affinity, site-limited uptake dominated by reactions at MgO/Mg(OH)_2_ surface sites rather than purely non-specific physical adsorption.^22,32,46,48,51,54^ In our system, the SEM-EDS observations of a transition from a C-rich, essentially smooth biochar surface to a Mg-rich, highly textured MgO-BC surface provide direct evidence of changes in structure and composition that support the interpretation of enhanced phosphate removal as MgO-mediated processes *i.e.*, specific chemisorption and surface precipitation on MgO/Mg(OH)_2_ sites dispersed on the biochar matrix, as opposed to non-specific adsorption on the original carbon surface.^55,56^

#### 3.1.7. XPS analysis

The surface elemental compositions and chemical states of WB-BC and MgO-BC were examined by XPS. The survey spectra (Fig. 5a and b) reveal that WB-BC is dominated by carbon (C 1s, ≈88.8 at%) with minor oxygen (O 1s, ≈9.9 at%) and trace Ca 2p (≈1.3 at%), consistent with the largely carbonaceous nature of lignocellulosic biochar pyrolyzed at 600 °C. In contrast, the MgO-BC survey spectrum shows a prominent Mg KLL Auger feature, a greatly intensified O 1s signal and a distinct Mg 2p peak (≈9.4 at%), while the relative C 1s contribution decreases to = 62.0 at% (O 1s ≈ 28.6 at%), confirming the successful surface incorporation of Mg-containing phases during MgCl_2_ impregnation and co-pyrolysis. Deconvolution of the C 1s spectra (Fig. 6a and c) reveals four components for both materials: the dominant peak at 284.7–284.8 eV (C–C/C–H, sp^2^ graphitic and aliphatic carbon, ≈68–81% of total C 1s area), a shoulder at 286.3–286.5 eV (C–O, phenolic/ether), a component near 288.5–288.7 eV (C=O, carbonyl/carboxyl), and a minor contribution at ≈290.3–290.9 eV (O–C=O, carboxylate or carbonate), all in agreement with established C 1s reference values for biochar. The relative area of the oxygenated C–O and O–C=O components is modestly higher in MgO-BC, indicating that Mg modification enriches surface oxygen functionalities, consistent with previous reports on Mg-impregnated biochars. The O 1s region (Fig. 6b and d) of WB-BC is fitted with three components: a lower-binding-energy peak at = 531.2 eV (C=O in carbonyl/quinone environments), a dominant contribution at ≈531.6 eV (C–O, phenolic/ether, 10.5% of O 1s), and a major peak at ≈533.2 eV (carboxylate/ester oxygen, 63.2%), consistent with oxygenated surface groups on biochar. For MgO-BC, the O 1s envelope narrows and is dominated by two components centered at ≈532.1 eV (=29.9%) and ≈533.9 eV (≈70.1%), corresponding to lattice oxygen in MgO/Mg-oxide phases and hydroxyl oxygen in Mg(OH)_2_ and surface carbonate environments, respectively, in agreement with published O 1s reference values for MgO and Mg(OH)_2_ on carbon supports. The Mg 2p spectrum of MgO-BC (Fig. 6e) is resolved into two components at 49.9 eV (≈47% area) and 52.1 eV (≈53% area). The lower-binding-energy component is consistent with MgO lattice Mg^2+^ (∼49.3–49.6 eV), while the higher-binding-energy contribution is characteristic of Mg(OH)_2_ or basic magnesium carbonate (≈50.5–50.7 eV), demonstrating the coexistence of oxide and hydroxide Mg phases on the biochar surface. Collectively, the XPS data confirm that MgO modification converts the predominantly carbonaceous surface of WB-BC into an MgO/Mg(OH)_2_-enriched surface with elevated oxygen content and active Mg–O/Mg–OH sites, which are the primary reactive centers responsible for inner-sphere phosphate complexation and surface precipitation.

**Fig. 5:**
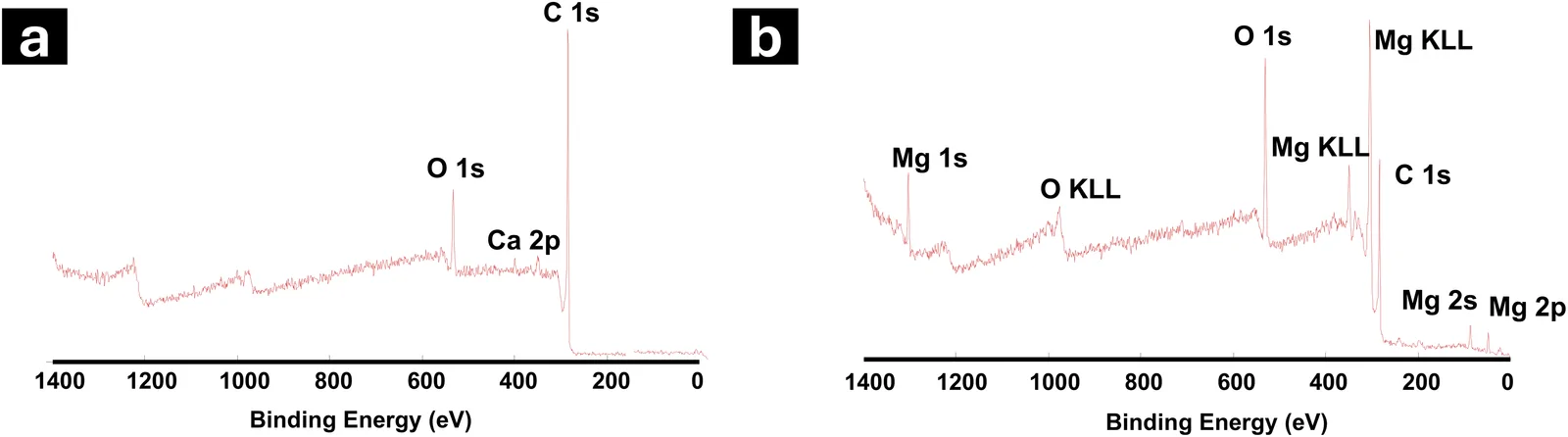
XPS survey spectra of pristine wattle bark biochar (WB-BC) and MgO-modified biochar (MgO-BC): (a) WB-BC and (b) MgO-BC.

**Fig. 6:**
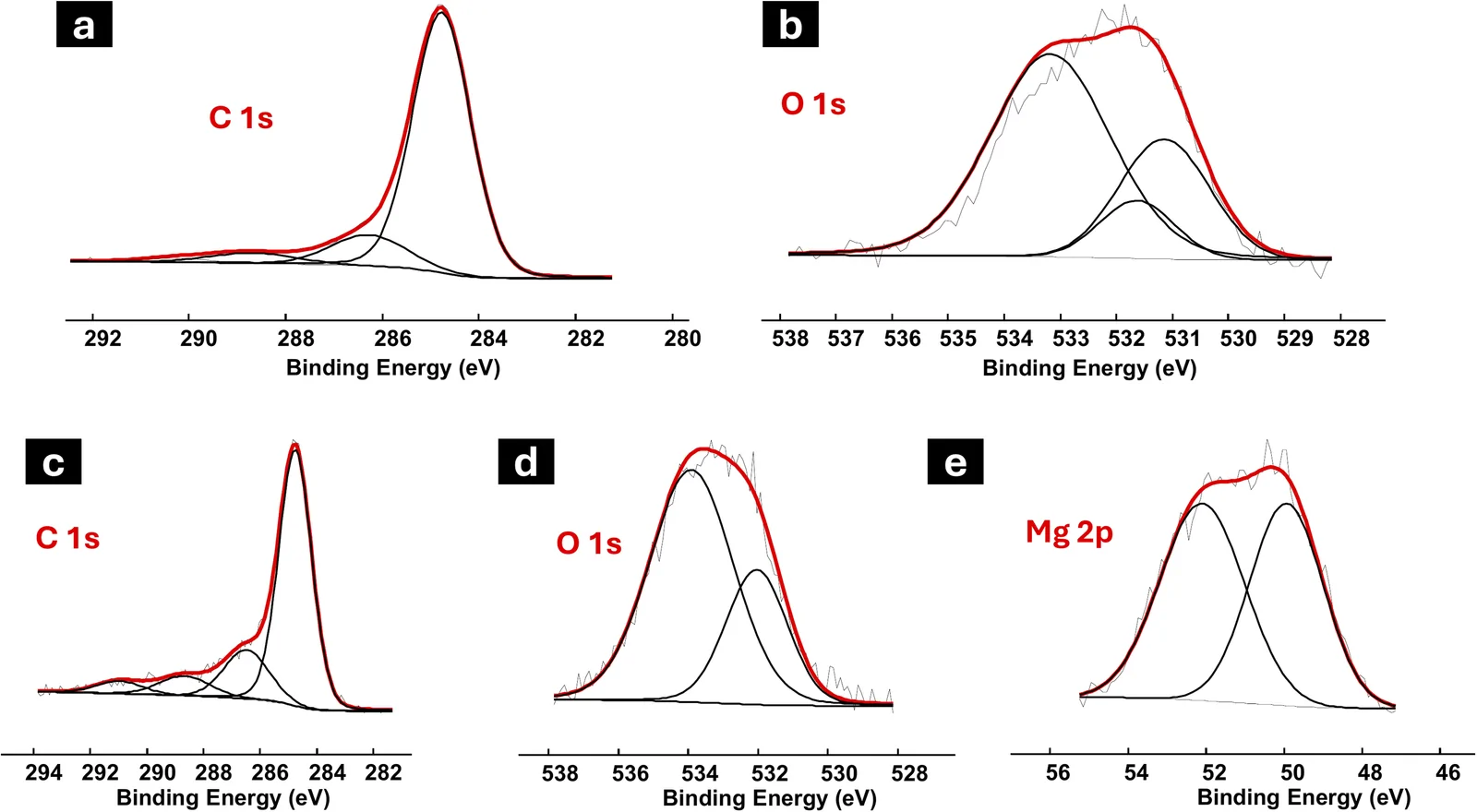
High-resolution XPS spectra of WB-BC and MgO-BC: (a) C 1s of WB-BC, (b) O 1s of WB-BC, (c) C 1s of MgO-BC, (d) O 1s of MgO-BC, and (e) Mg 2p of MgO-BC. Peak deconvolution highlights the carbonaceous surface functionalities of WB-BC and the presence of MgO/Mg(OH)_2_-related species on MgO-BC.

To further investigate phosphate interaction with MgO-BC, XPS analysis was performed after adsorption (Fig. 7). The survey spectrum of phosphate-loaded MgO-BC shows O, C, and Mg signals together with a weak but detectable P 2p contribution of 0.4 at%, confirming phosphorus retention at the adsorbent surface. The post-adsorption survey quantification yielded O 1s = 47.3 at%, C 1s = 34.0 at%, Mg 1s = 18.3 at%, and P 2p = 0.4 at%. Because Mg was quantified from the Mg 1s signal in the post-adsorption survey whereas Mg 2p was used for the fresh MgO-BC survey, the Mg atomic percentages before and after adsorption should not be directly compared quantitatively.

**Fig. 7:**
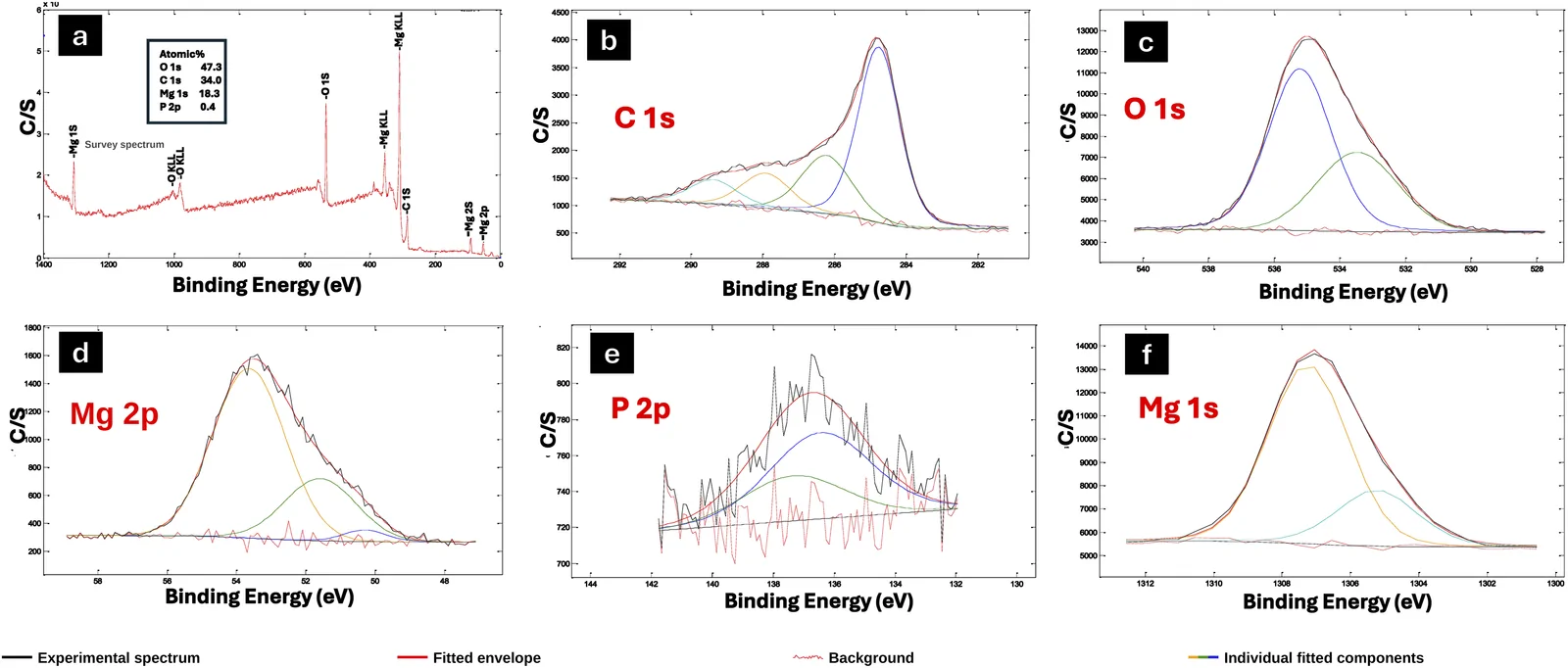
XPS spectra of MgO-BC after phosphate adsorption: (a) survey spectrum with surface elemental composition; high-resolution spectra of (b) C 1s, (c) O 1s, (d) Mg 2p, (e) P 2p, and (f) Mg 1s. The phosphate-loaded MgO-BC was obtained under the optimized conditions: pH 8.0, initial phosphate concentration *C*_0_ = 64.6 mg P per L, MgO-BC dosage of 0.059 g in 10 mL solution, contact time of 21 h, and temperature of 25 °C. The weak but detectable P 2p signal confirms phosphate retention on the MgO-BC surface. Black, red, dashed, and coloured curves denote the experimental spectrum, fitted envelope, background, and individual fitted components, respectively.

The weak P 2p envelope centered in the approximately 136–137 eV range is consistent with oxidized phosphate species retained at the MgO-BC surface. Compared with fresh MgO-BC, the O 1s envelope of the phosphate-loaded material changes after adsorption, indicating the involvement of oxygen-containing Mg–O/Mg–OH surface sites and/or surface hydroxyl groups in phosphate uptake. The Mg 2p envelope is also broadened after adsorption and requires an additional higher-binding-energy fitted contribution, demonstrating a modified local Mg chemical environment after contact with phosphate. The concurrent detection of P 2p and changes in the O 1s and Mg 2p regions provide direct surface-sensitive evidence of phosphate interaction with Mg-containing surface sites.

In combination with the negligible ionic-strength effect and the absence of newly resolved sharp magnesium-phosphate reflections in the XRD pattern, the post-adsorption XPS results support a mechanism dominated by Mg-centered inner-sphere complexation, accompanied by poorly crystalline or amorphous surface precipitation rather than bulk formation of a well-crystallized magnesium-phosphate phase.

### 3.2. Central composite design and response surface analysis

A five-level, four-factor central composite design (CCD) was used to study the adsorption of phosphate (*R*%) by MgO-modified wattle bark biochar (MgO-BC). The investigated factors include solution pH (*A*, 3.5–11.0), MgO-BC dosage (*B*, 0.001–0.100 g), contact time (*C*, 8–24 h), and initial phosphate concentration (*D*, 5–100 mg L^−1^), each studied at five coded levels (−2, −1, 0, +1, +2), as summarized in Table 2. The CCD contact-time factor (*C*, 8–24 h) was deliberately set wide enough to guarantee equilibrium attainment across the entire multi-factor design space, since dosage and concentration were varied simultaneously with time. Independent kinetic experiments (Section 3.4) show that phosphate uptake on MgO-BC is fast, with >95% of the 360 min equilibrium capacity attained within 120 min (Fig. 10b). Consequently, the 8–24 h CCD range lies almost entirely on the post-equilibrium plateau, which explains the comparatively minor main effect of contact time relative to dosage and initial concentration in the quadratic model (Table 4). The true rate-limiting kinetic window (0–120 min) is instead resolved by the pseudo-second-order model in Section 3.4, and should be consulted for process-design purposes rather than the CCD time range itself. The observed *R*% values ranged from 75.49 to 99.94% (mean: 94.21%), indicating high removal efficiency across the design space with sufficient variance for robust model. Based on fit summary statistics and sequential *F*-tests, a quadratic polynomial model was selected as the best fit. This model demonstrated significant improvements over a linear and two-factor interaction models, with a non-significant lack-of-fit. The quadratic model yielded *R*^2^, adjusted *R*^2^ and predicted *R*^2^ values of 0.9919, 0.9843 and 0.9694, respectively, confirming excellent predictive capacity for response-surface optimization.

**Table 2:** Experimental factors and levels used in the central composite design (CCD)

Factor	Variable	Units	Minimum	Maximum	Coded low (−1)	Coded high (+1)	Centre (0, mean)
*A*	pH	—	3.50	11.00	4.60	9.90	7.25
*B*	MgO-biochar dosage	g	0.0010	0.1000	0.0200	0.0900	0.0505
*C*	Contact time	h	8.00	24.00	10.34	21.66	16.00
*D*	Initial phosphate conc.	mg L^−1^	5.00	100.00	18.91	86.09	52.50

Such high removal efficiencies over a broad design space are consistent with previous CCD/RSM studies on phosphate adsorption, where Mg-modified biochars and granular ferric or lanthanum-based sorbents typically achieve removals above 80–90% under optimized conditions.^57–59^

The sequential model sum-of-squares, fit summary and lack-of-fit tests confirm that a quadratic polynomial was the most suitable model for *R*%. The quadratic *vs.* two-factor interaction (2FI) was highly significant (*F* = 234.48, *p* < 0.0001), quadratic terms to account for the system's inherent curvature. While the linear and 2FI models exhibit significant lack-of-fit (*p* < 0.0001), the quadratic model showed non-significant lack-of-fit (*F* = 3.01, *p* = 0.1175), indicating its adequacy in describing the experimental data. The model yielded high determination coefficients for the quadratic model were *R*^2^ = 0.9919, adjusted *R*^2^ = 0.9843 and predicted *R*^2^ = 0.9694, with low prediction error sum of squares (PRESS = 33.19) and minimal coefficient of variation (0.81%). Collectively, these parameters demonstrate high descriptive and predictive power, comparing favorably with recent CCD-based optimizations of phosphate removal *via* adsorption and electrochemistry.^60,61^

ANOVA for the quadratic model (Table 3) showed that the regression was highly significant (*F* = 130.97, *p* < 0.0001), while the lack-of-fit was not significant, showing adequacy of the model in the explored design space. The linear terms MgO-BC dosage (*B*) and initial phosphate concentration (*D*) were highly significant (*p* < 0.0001), demonstrating that the number of reactive sites on MgO-BC and the initial mass-transfer driving force are the main direct factors influencing *R*_1_. Although removal efficiency is concentration-dependent by definition, it is retained here as the optimization response because the operational target in phosphate polishing is achieving a specified effluent quality. The corresponding equilibrium uptake under the optimized conditions was evaluated separately from the adsorption experiments. The Langmuir maximum adsorption capacity obtained from the extended isotherm analysis was *q*_max_ = 11.42 ± 0.11 mg P g^−1^ (Section 3.4); this fitted parameter should be distinguished from the experimentally measured *q*_e_ at any individual adsorption condition. Conversely, the linear terms for pH (*A*) and contact time (*C*) were not significant at the 95% level (*p* = 0.0701 and 0.1758, respectively), showing that their effects are mostly expressed through quadratic and/or interaction terms, rather than monotonic tendencies. This is consistent with other CCD studies of phosphate and other oxyanion removal, where adsorbent dosage and initial concentration are typically the most significant terms, whereas pH and contact time affect the efficiency in more complex ways.^58,62,63^ The significant interaction terms *AB*, *AD*, *BD* and *CD* (*p* < 0.05) indicate that the influence of pH and contact time is dependent on dosage and initial concentration, and that multiple factors need to be considered in the optimization process instead of the one-factor-at-a-time method.^64^ The quadratic terms *A*^2^, *B*^2^, *C*^2^ and *D*^2^ were significant (*p* ≤ 0.0175), with *B*^2^ showing the highest *F*-value (884.13), revealing a strong curvature, especially for the MgO-BC dosage (Table 4). The fitted quadratic model in coded variables was:*R* = 97.34 − 0.3341*A* + 4.36*B* + 0.2321*C* + 2.15*D* + 1.50*AB* + 0.2121*AC* − 0.5425*AD* − 0.0754*BC* − 0.5375*BD* − 0.5020*CD* + 1.64 *A*^2^ − 7.50*B*^2^ + 1.67*C*^2^ − 0.6734*D*^2^.

**Table 3:** Analysis of variance (ANOVA) for the quadratic model of phosphate removal (*R*_1_)^a^

Source	Sum of squares	d*f*	Mean square	*F*-Value	*p*-Value	
Model	1076.20	14	76.87	130.97	<0.0001	Significant
*A*-PH	2.23	1	2.23	3.80	0.0701	
*B*-MgO dosage	379.33	1	379.33	646.28	<0.0001	
*C*-Time	1.18	1	1.18	2.02	0.1758	
*D*-Phosphate concentration	92.41	1	92.41	157.44	<0.0001	
*AB*	35.76	1	35.76	60.93	<0.0001	
*AC*	0.8100	1	0.8100	1.38	0.2584	
*AD*	4.71	1	4.71	8.02	0.0126	
*BC*	0.1024	1	0.1024	0.1745	0.6821	
*BD*	4.62	1	4.62	7.88	0.0133	
*CD*	4.54	1	4.54	7.73	0.0140	
*A* ^2^	24.84	1	24.84	42.32	<0.0001	
*B* ^2^	518.93	1	518.93	884.13	<0.0001	
*C* ^2^	27.99	1	27.99	47.68	<0.0001	
*D* ^2^	4.18	1	4.18	7.12	0.0175	
Residual	8.80	15	0.5869			
Lack of fit	7.55	10	0.7551	3.01	0.1175	Not significant
Pure error	1.25	5	0.2505			
Corrected total	1085.01	29				

a Model statistics: *R*^2^ = 0.9919; adjusted *R*^2^ = 0.9843; predicted *R*^2^ = 0.9694; std. dev. = 0.7661; PRESS = 33.19.

**Table 4:** Regression coefficients of the quadratic model in coded variables for phosphate removal (*R*%)

Factor	Coefficient estimate	df	Standard error	95% CI low	95% CI high	VIF
Intercept	97.34	1	0.2727	96.76	97.92	
*A*-PH	−0.3341	1	0.1713	−0.6993	0.0310	1.0000
*B*-MgO dosage	4.36	1	0.1713	3.99	4.72	1.0000
*C*-Time	0.2321	1	0.1633	−0.1161	0.5802	1.0000
*D*-Phosphate concentration	2.15	1	0.1713	1.78	2.51	1.0000
*AB*	1.50	1	0.1915	1.09	1.90	1.0000
*AC*	0.2121	1	0.1806	−0.1728	0.5970	1.0000
*AD*	−0.5425	1	0.1915	−0.9507	−0.1343	1.0000
*BC*	−0.0754	1	0.1806	−0.4603	0.3095	1.0000
*BD*	−0.5375	1	0.1915	−0.9457	−0.1293	1.0000
*CD*	−0.5020	1	0.1806	−0.8869	−0.1172	1.0000
*A* ^2^	1.64	1	0.2523	1.10	2.18	1.16
*B* ^2^	−7.50	1	0.2523	−8.04	−6.97	1.16
*C* ^2^	1.67	1	0.2419	1.15	2.19	1.21
*D* ^2^	−0.6734	1	0.2523	−1.21	−0.1355	1.16

The positive linear coefficients for MgO-BC dosage and initial phosphate concentration indicate that these factors contribute significantly to the fitted response within the tested design space. However, because percentage removal is intrinsically dependent on the ratio of solute loading to available adsorbent sites, the effect of initial phosphate concentration must be interpreted together with the significant quadratic and interaction terms rather than as a simple monotonic increase in removal efficiency. At a fixed MgO-BC dosage, increasing the initial phosphate concentration ultimately decreases percentage removal as the finite population of high-affinity Mg-containing sites becomes progressively saturated. This reflects an increase in available MgO/Mg(OH)_2_ active sites and a stronger concentration gradient driving diffusion to the sorbent surface, respectively. Conversely, the significantly negative quadratic coefficient for dosage (*B*^2^ = −7.50) indicates an optimum MgO-BC dosage threshold. Beyond this point, further increases in MgO-BC lead to a decline in percentage removal. This is likely attributed to particle aggregation and site overlap, which reduces the specific surface area per unit mass of sorbent. Such behavior has been reported in RSM studies on Mg- and Ca-modified biochar and granular ferric sorbents, in which overdosing leads to lower percentage removal (but higher absolute uptake).^22,65–68^ The coefficients of *A*^2^, *C*^2^ and *D*^2^, suggest closed optima for pH, contact time and initial concentration, representing the equilibrium between charge-surface interactions, diffusion time and saturation of high-affinity sites. Variance Inflation Factors (VIF) were near unity, suggesting that there was minimal multicollinearity, ensuring that the effects of individual factors were accurately estimated (Table 4).^69–71^

Assessment of the model further confirmed the quadratic regression. The normal probability plot of externally studentized residuals exhibited a linear distribution with only minor deviations at tails, showing that the residuals followed a near-normal distribution (Fig. 8a). Residuals *versus* predicted values (Fig. 8b) were randomly distributed around zero, with no evidence of funneling or other systematic patterns. This satisfies the condition of homoscedasticity and indicates the absence of model misspecification within the design space. All externally studentized residuals remained within the ±4 limits (Fig. S1a) and all Cook's distances were much smaller than 1.0 (Fig. S1b). These results confirm that no individual data point had an inordinate influence on the fitted quadratic model, adhering to established best practices for CCD/RSM modelling for adsorption and water-treatment applications.^72–74^

**Fig. 8:**
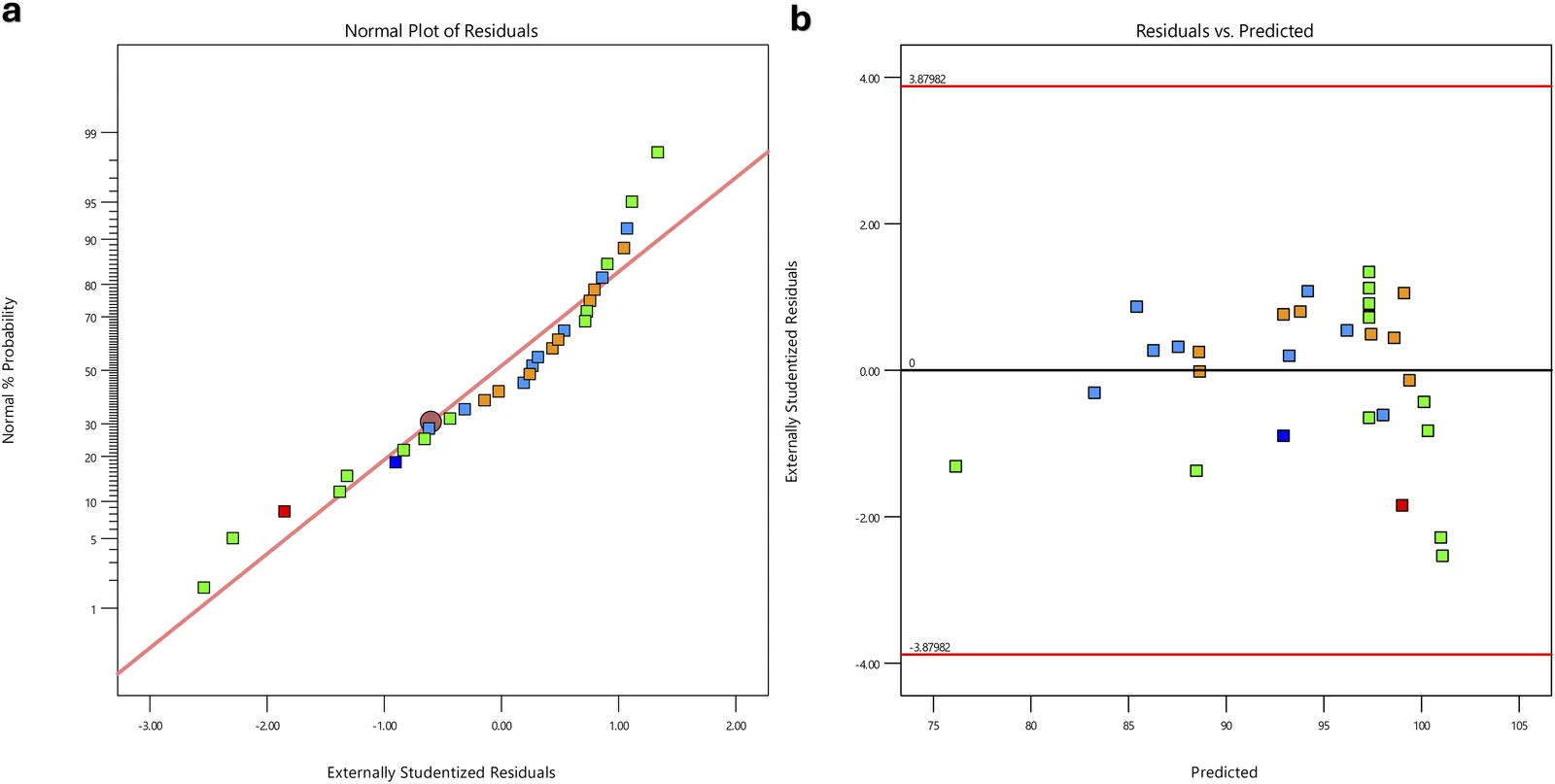
(a) Normal probability plot of externally studentized residuals for the quadratic model of phosphate removal, showing approximate normality of the errors. (b) Plot of externally studentized residuals *versus* predicted phosphate removal, indicating homoscedasticity and absence of systematic patterns within the design space.

Numerical optimization was performed using the desirability function to maximize phosphate removal (*R*%) over all four factors. The software identified a region of equivalent optimal solutions with an overall desirability of 1.0 and predicted removals close to 100%. A convenient optimum was selected at pH 8.05, MgO-BC dosage 0.059 g, contact time 20.98 h and initial phosphate concentration 64.61 mg L^−1^, corresponding to a predicted *R*% of 100.06 (desirability = 1.000) (Table 5). This optimum at moderately alkaline pH, intermediate MgO-BC dose and medium initial concentration is in good agreement with CCD-based optimization studies of Mg-modified biochars and Fe- or La-based phosphate sorbents, which also report maximum removal under conditions where the sorbent surface is positively charged but not overdosed and the pollutant loading is moderate.^63^ These articles typically report peak phosphate removal in regions where the sorbent surface is positively charged but not overloaded and where the number of binding sites and loading of the pollutant are balanced. Experimental verification at these conditions was in good agreement with the model predictions, suggesting that the quadratic model can be used to explore the design space and to identify conditions that are likely to yield the highest phosphate removal under practical water-treatment conditions.^57,58,65,75^

**Table 5:** Selected optimal conditions obtained from numerical desirability optimization for maximizing phosphate removal (*R*%)

Solution	pH	MgO-biochar dosage (g)	Contact time (h)	Initial phosphate conc. (mg L^−1^)	Predicted *R*_1_ (%)	Overall desirability
Selected	8.05	0.059	20.98	64.61	100	1.000

### 3.3. Interactive effects and mechanistic interpretation

The three-dimensional response surface of pH and MgO-biochar dosage in Fig. 9a indicates an increase in phosphate removal with higher dosages across the tested pH range, with the most pronounced gradient occurring at slightly alkaline conditions (pH 8). This trend aligns with the enhancement of electrostatic attraction between divalent (HPO_4_^2−^) or monovalent phosphate species (H_2_PO_4_^−^) and the positively charged MgO/Mg(OH)_2_-enriched surfaces near and below the pH_PZC_, as well as the increased density of surface sites at higher sorbent dosages. At lower dosages, the scarcity of active Mg–OH and Mg–O surface groups limits removal efficiency, whereas increasing the dosage increases the collision rate between ions and sorbent, which raises the likelihood of binding, a behavior typical of Mg-modified biochar and other metal-oxide sorbents for phosphate removal. The slight decrease or plateau in *R* at very high MgO-biochar dosages (reflected by the strongly negative quadratic term *B*^2^) is in line with particle aggregation and site overlapping, which effectively decrease the specific surface area per sorbent mass, as documented in other RSM-optimized adsorption systems.^76–79^ As shown in Fig. 9a, phosphate removal by MgO-BC increases sharply when the initial pH is raised from strongly acidic to near-neutral conditions at a given MgO-BC dosage and remains above 95% in the pH 6–9 range under the investigated conditions. This trend reflects the combined effects of phosphate speciation and surface charge. At low pH, the dominant H_2_PO_4_^−^ species competes with protons for partially protonated Mg–OH/Mg–O surface sites, which limits uptake, whereas at near-neutral pH the increased fraction of HPO_4_^2−^ and the strongly positive MgO-BC surface (pH < pH_PZC_ = 10.4; Section 3.1.2) enhance electrostatic attraction and facilitate inner-sphere complexation at Mg-rich centers. At pH values above 9, the removal efficiency shows a slight decrease or plateau, which can be attributed to the progressive approach to the pH_PZC_ and increased competition from OH^−^ anions. From an application perspective, the ability of MgO-BC to sustain >95% phosphate removal over pH 6–9, a range that encompasses most municipal and agricultural wastewaters, suggests that effective treatment can be achieved without extensive pH adjustment in many real-water matrices.

**Fig. 9:**
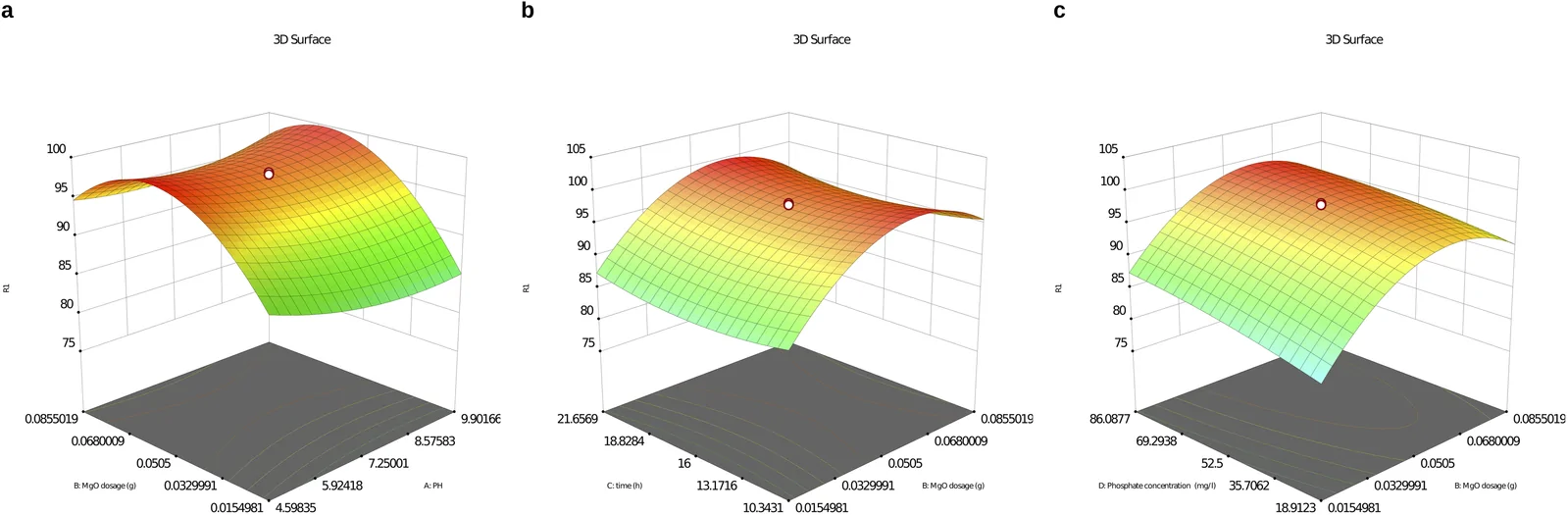
Three-dimensional response surface and contour plots for phosphate removal (*R*%) by MgO-modified wattle bark biochar as a function of (a) pH and MgO-biochar dosage (A and B), (b) MgO-biochar dosage and contact time (B and C) and (c) MgO-biochar dosage and initial phosphate concentration (B–D), with the remaining variables fixed at their central levels. The plots reveal pronounced positive effects of MgO-biochar dosage and initial phosphate concentration, significant A–B and B–D interactions, and well-defined curvature of the response, in agreement with the quadratic CCD/RSM model and with similar adsorption optimization studies.

**Fig. 10:**
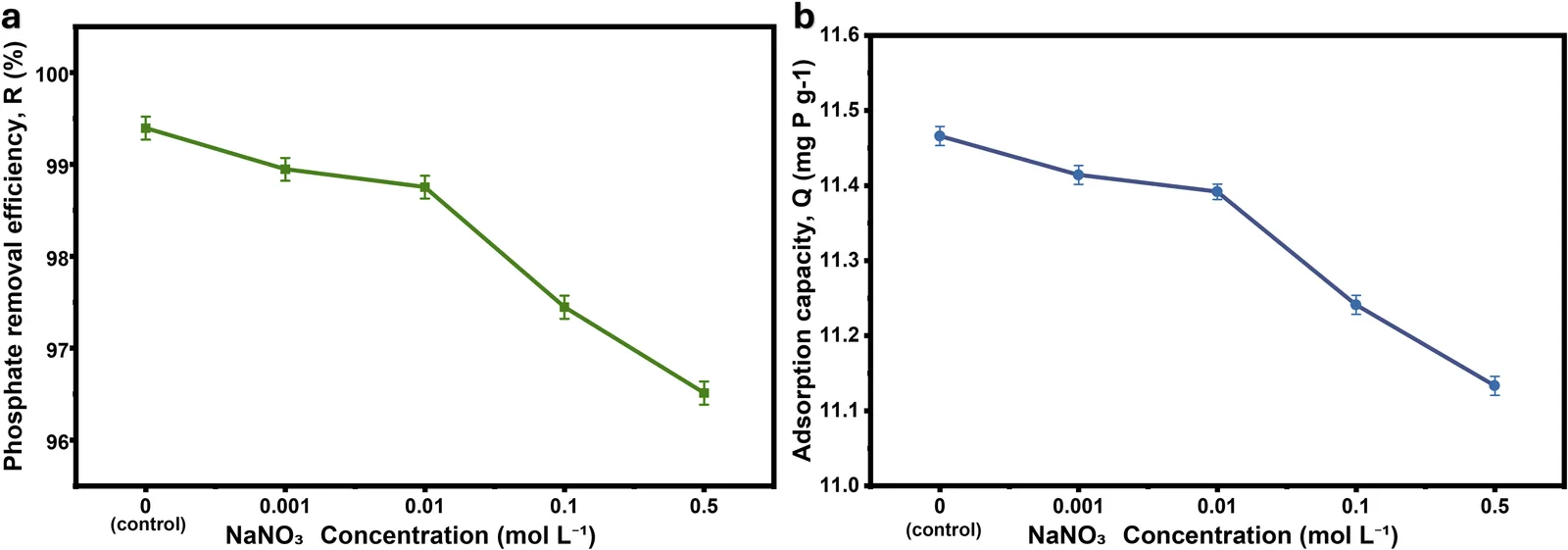
Effect of NaNO_3_ ionic strength on phosphate uptake by MgO BC. (a) Phosphate removal efficiency, *R* (%), and (b) equilibrium adsorption capacity, *q*_e_ (mg P g^−1^), as a function of NaNO_3_ concentration (0–0.5 mol L^−1^). Error bars represent standard deviations (*n* = 3). Experimental conditions: *C*_0_ = 64.61 mg P per L, pH 8.05, MgO BC dosage 0.059 g per 10 mL solution, contact time 21 h, *T* = 25 °C.

The interaction surface between dosage and contact time (Fig. 9b) suggests that phosphate removal is more sensitive to sorbent dosage than to contact time in the investigated region. Efficiency (*R*) reaches a plateau between 16 and 21 h at higher dosages, indicating that film and intraparticle diffusion resistance are largely eliminated within this time frame. Consequently, the overall adsorption process is constrained by the availability of high-affinity binding sites rather than by very slow diffusion kinetics, an observation consistent with pseudo-second-order behavior typically reported for metal-oxide-modified biochar and other nanostructured sorbents. The modest linear and pronounced quadratic response to contact time can be explained by a mechanism characterized by rapid surface complexation onto Mg–OH/Mg–O sites, followed by a slower relaxation towards equilibrium, with negligible gains in percentage removal beyond the optimum contact time.^80^

The response surface for initial phosphate concentration and MgO-biochar dosage (Fig. 9c) shows that percentage removal reaches a maximum for intermediate concentrations and moderate-to-high dosages. High removal (at the expense of absolute uptake) is achieved at very low concentrations, whereas a slight decrease in percentage removal occurs at very high concentrations due to saturation of high-affinity sites. This is typical of Langmuir-type mechanisms, where saturation of a finite number of energetically equivalent sites is approached as the mass-transfer driving force increases. Such trends are well documented for phosphate removal using MgO-biochar, γ-Al_2_O_3_ and various metal–organic-framework- and graphene-oxide-based adsorbents. Consequently, the interplay between dosage and initial phosphate concentration represents a balance between maximizing the concentration gradient and maintaining sufficient capacity within the CCD design space. The curvature and interaction effects observed in Fig. 8 are consistent with the significant *B*, *D*, *BD* and *B*^2^ terms in the quadratic model, corroborating a mechanism dominated by surface complexation and electrostatic attraction at Mg-rich sites, as reported for the isotherm and kinetic data of similar phosphate-adsorbent systems.^81,82^

#### 3.3.1. Effect of ionic strength

The influence of ionic strength on phosphate removal by MgO-BC is shown in Fig. 10. Phosphate removal efficiency remained high across all NaNO_3_ concentrations tested, decreasing only slightly from 99.4 ± 0.5% at 0 mol L^−1^ to 96.5 ± 0.6% at 0.5 mol L^−1^ NaNO_3_ (Fig. 10a). A corresponding minor decrease in equilibrium adsorption capacity from 11.47 ± 0.06 to 11.13 ± 0.10 mg P g^−1^ was observed (Fig. 10b). These small changes, less than 3 percentage points in *R* and less than 0.35 mg P g^−1^ in *q*_e_ over three orders of magnitude increase in ionic strength, demonstrate that background electrolyte exerts only a minor influence on phosphate uptake under the conditions studied. Across the NaNO_3_ range 0–0.5 mol L^−1^, phosphate removal decreased by less than 3 percentage points and *q*_e_ decreased by less than 0.35 mg P g^−1^, indicating that the adsorption is largely insensitive to ionic strength and is therefore governed by inner-sphere complexation and surface precipitation rather than outer-sphere electrostatic interaction.

### 3.4. Adsorption behavior and mechanism (isotherms and kinetics)

The equilibrium adsorption of phosphate onto MgO-BC was investigated over an extended initial concentration range (*C*_0_ = 15–500 mg P per L; *C*_e_ = 0.013–432.3 mg P per L) at pH 8.0 with a constant adsorbent dose (0.059 g in 10 mL) and 21 h contact time. Non-linear regression of the Langmuir, Freundlich and Temkin models to the *C*_e_ − *q*_e_ data (Fig. 11a) was performed in OriginPro using user-defined functions, and goodness-of-fit was assessed using the coefficient of determination (*R*^2^), adjusted *R*^2^ and reduced chi-squared (*χ*_red._^2^); the resulting parameters are summarized in Table 6.

**Fig. 11:**
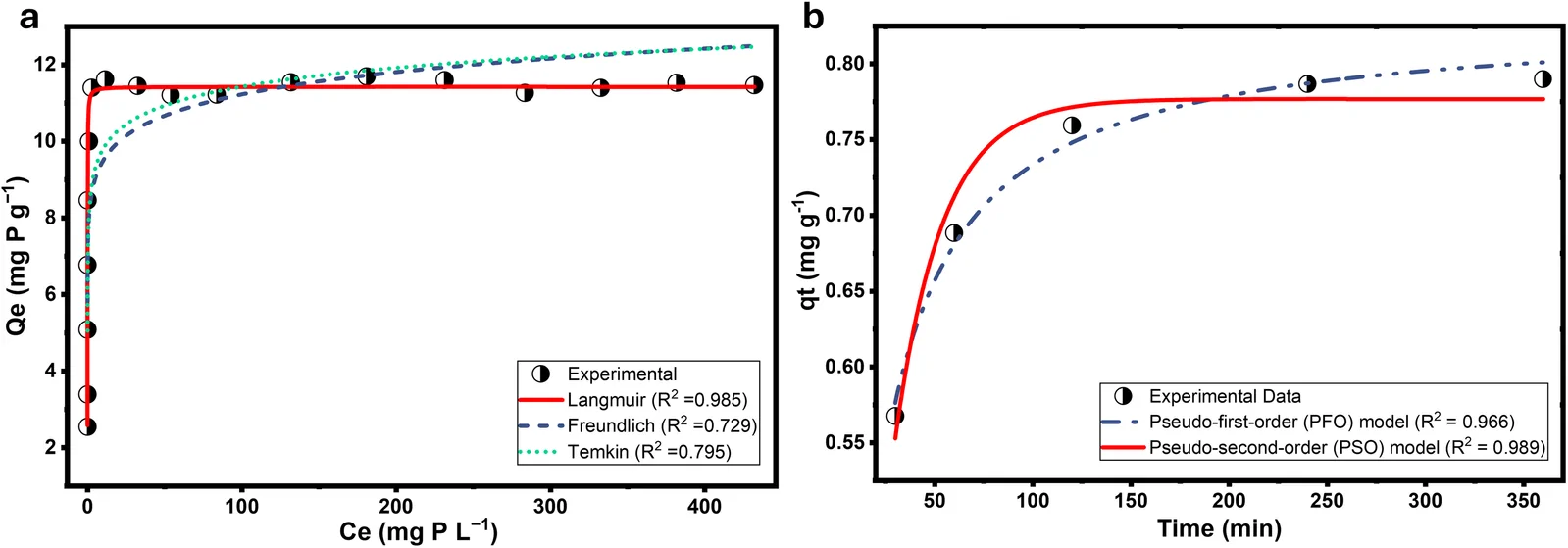
(a) Non-linear Langmuir, Freundlich and Temkin isotherm fits for phosphate adsorption onto MgO-BC at 298 K; symbols represent experimental equilibrium data and lines denote model predictions. (b) Non-linear pseudo-second-order (PSO) and pseudo-first-order (PFO) kinetic fits for phosphate adsorption onto MgO-BC; symbols denote experimental *q*_*t*_ values and lines show the corresponding model simulations, demonstrating the superior agreement of the Langmuir and PSO models with the experimental data. Fixed conditions: MgO BC dosage 0.059 g in 10 mL solution; pH 8.0; temperature 25 °C. (a) Initial phosphate concentration range 15–500 mg P per L; contact time 21 h. (b) Initial phosphate concentration 64.6 mg P per L; contact time 0–360 min.

**Table 6:** Parameters of the non-linear Langmuir, Freundlich and Temkin isotherm models for phosphate adsorption onto MgO-BC at 298^a^

Model	Parameter	Value
Langmuir isotherm	*q*_max_ (mg P per g)	11.42 ± 0.11
	*K*_L_ (L mg^−1^)	22.43 ± 1.58
	*R*_L_	8.9 × 10^−5^–2.96 × 10^−3^
	*R* ^2^/*χ*_red._^2^	0.985/0.146
Freundlich isotherm	*K*_F_ (mg g^−1^ (L mg^−1^)^1/*n*^)	8.01 ± 0.51
	*N*	13.66 ± 2.51
	*R* ^2^/*χ*_red._^2^	0.729/2.706
Temkin isotherm	*A*_T_ (L g^−1^)	(9.42 ± 1.69) × 10^4^
	*B* (mg g^−1^)	0.712 ± 0.090
	*b*_T_ (J mol^−1^)	3483
	*R* ^2^/*χ*_red._^2^	0.795/2.046
PSO kinetics	*q*_e_ (mg P per g)	0.830 ± 0.010
	*k*_2_ (g mg^−1^ min^−1^)	0.0909 ± 0.0081
	*R* ^2^/adj. *R*^2^/*χ*_red._^2^	0.989/0.985/1.31 × 10^−4^
PFO kinetics	*q*_e_ (mg P per g)	0.777 ± 0.012
	*k*_1_ (min^−1^)	0.0414 ± 0.0031
	*R* ^2^/adj. *R*^2^/*χ*_red._^2^	0.966/0.954/4.01 × 10^−4^

a *n* = 18 data points; values are mean ± standard error from non-linear regression in OriginPro.

The Langmuir model assumes monolayer adsorption on a finite number of energetically homogeneous and non-interacting sites, and yielded a maximum capacity *q*_max_ = 11.42 ± 0.11 mg P per g and affinity constant *K*_L_ = 22.43 ± 1.58 L mg^−1^ (*R*^2^ = 0.985, *χ*_red._^2^ = 0.146), indicating an excellent description of the equilibrium data. The corresponding dimensionless separation factor *R*_L_ = 1/(1 + *K*_L_*C*_0_) was 8.9 × 10^−5^–2.96 × 10^−3^ over the investigated *C*_0_ range, *i.e.* 0 < *R*_L_ ≪ 1, confirming that phosphate adsorption onto MgO-BC is highly favorable and approaches irreversibility at higher concentrations. The derived *q*_max_ falls within the capacity range reported for Mg-modified biochars prepared by simple impregnation routes, while the comparatively high *K*_L_ highlights the strong affinity of phosphate for Mg–O/Mg–OH surface domains, which is particularly beneficial for polishing effluents to low residual P levels.

The Freundlich isotherm, which empirically describes adsorption on heterogeneous surfaces, produced *K*_F_ = 8.01 ± 0.51 mg g^−1^ (L mg^−1^)^1/*n*^ and *n* = 13.66 ± 2.51 (*R*^2^ = 0.729), indicating highly favourable adsorption (*n* > 1) but a significantly poorer fit than the Langmuir model. The Temkin isotherm, accounting for a linear decrease in adsorption heat with coverage, gave *A*_T_ = 9.42 × 10^4^ ± 1.69 × 10^5^ L g^−1^ and *B* = 0.712 ± 0.090 (*R*^2^ = 0.795), corresponding to a heat-related constant *b*_T_ = *RT*/*B* = 3483 J mol^−1^, but with large relative uncertainty in *A*_T_ and inferior overall statistics compared with the Langmuir equation. Collectively, these results demonstrate that the Langmuir model most accurately describes phosphate adsorption on MgO-BC, supporting adsorption on a finite population of high-affinity Mg–O/Mg–OH sites, in agreement with the high pH_PZC_, FTIR/XRD evidence for MgO/Mg(OH)_2_ domains, and the absence of new well-crystalline Mg-phosphate phases after adsorption.

The time-dependent uptake of phosphate at *C*_0_ = 10 mg P per L exhibited a rapid initial stage during the first 60–120 min, followed by a slower approach to equilibrium at 360 min (Fig. 11b). Non-linear fitting of the kinetic data to pseudo-second-order (PSO) and pseudo-first-order (PFO) models showed that the PSO equation provides the superior description, yielding *q*_e_ = 0.830 ± 0.010 mg P per g and *k*_2_ = 0.0909 ± 0.0081 g mg^−1^ min^−1^ (*R*^2^ = 0.989, *χ*^2^ = 1.31 × 10^−4^), whereas the PFO model gave *q*_e_ = 0.777 ± 0.012 mg P per g and *k*_1_ = 0.0414 ± 0.0031 min^−1^ with lower statistical quality and underestimation of the equilibrium capacity (*R*^2^ = 0.966, *χ*_red._^2^ = 4.01 × 10^−4^). The close agreement between the PSO-predicted and experimental *q*_e_ values, combined with the Langmuir isotherm and high *K*_L_, indicates that the overall rate is governed predominantly by surface reaction and site availability, consistent with a chemisorption-dominated mechanism involving inner-sphere complexation and surface precipitation at Mg-rich domains, as reported for other Mg-loaded biochars and MgO-based sorbents used for phosphate removal.

The weak dependence of *R* and *q*_e_ on NaNO_3_ concentration (0–0.5 mol L^−1^) provides strong evidence that phosphate adsorption on MgO-BC is governed primarily by inner-sphere complexation and surface precipitation at MgO/Mg(OH)_2_ sites, rather than by outer-sphere electrostatic attraction. In outer-sphere adsorption, increasing ionic strength compresses the diffuse double layer and promotes competition from background electrolyte ions, leading to a marked suppression of uptake, which is not observed here. This behavior is consistent with Mg-functionalized biochars and Ca/Mg-loaded biochars reported in the literature, where phosphate binds *via* specific Mg–O–P linkages and magnesium phosphate precipitation that are insensitive to moderate changes in ionic strength. Combined with the high pH_PZC_ (=10.4), Langmuir-type isotherms, pseudo-second-order kinetics, and XRD evidence for Mg-phosphate phases, the ionic-strength results confirm a chemisorption-dominated mechanism for phosphate removal on MgO-BC (Table 7).

**Table 7:** Comparison of MgO-modified biochars for phosphate removal reported in the literature^a^

Adsorbent	Precursor	*q*_max_ (mg P per g)	Isotherm	Kinetics	Optimum pH	Reusability (cycles, %)	Reference
Mg-loaded sludge biochar	Sewage sludge	109.35	Langmuir	PSO	∼7–8	ND	83
Mg-SCG-500	Spent coffee grounds	111.20	Langmuir	PSO	∼7	20 cycles (column)	45
MgO-BC (peanut shell)	Peanut shell	30.48–114.24	Langmuir	PSO	7–8	—	51
MgO-BC (rice straw)	Rice straw	24.71	Langmuir	PSO	∼7	ND	47
Mg-BC (hardwood)	Red oak/maple	28.20–29.22	Langmuir	PSO	∼5–7	ND	84
Fe/MgO-BC (*Acacia tortilis*)	*Acacia* pruning waste	98.50	Langmuir	PSO	8.5	ND	44
MgO-BC (ultrafine nanoparticles)	Unspecified biomass	High (nanoscale MgO)	Langmuir	PSO	∼7	5 cycles, good	46
MgO-BC (this work)	*Acacia mearnsii* bark	11.42	Langmuir	PSO	8.0	5 cycles, >80%	This work

a ND = not determined.

### 3.5. Powder XRD analysis before and after adsorption

X-ray diffractograms of MgO-BC before and after phosphate adsorption (Fig. 12a) are dominated by intense reflections characteristic of periclase-type MgO (M, PDF 45-0946) and brucite-type Mg(OH)_2_ (B, JCPDS 7-0239), confirming that the main crystalline phases formed during MgCl_2_ impregnation and pyrolysis at 600 °C are preserved after phosphate loading. The main diffraction peaks of pristine MgO-BC are indexed to periclase-MgO, with reflections at 2*θ* ≈ 36.9°, 42.8°, 62.2°, 74.6° and 78.5°, corresponding to the (111), (200), (220), (311) and (222) planes, respectively, in good agreement with values reported for structurally comparable MgO/biochar phosphate sorbents.^83^ Minor reflections at 2*θ* ≈ 37.9°, 50.7° and 58.6° are attributed to brucite-Mg(OH)_2_ (101), (102) and (110) planes, consistent with reference brucite diffraction data.^85^ Following phosphate adsorption, two additional weak brucite reflections become resolved at 2*θ* ≈ 18.6° (001) and 68.3°/72.0° ((200)/(201)). The principal MgO peaks, together with the brucite peaks, remain at essentially identical positions (Δ2*θ* < 0.1° for all indexed reflections) and with similar relative intensities after adsorption, and no new sharp reflections attributable to crystalline magnesium phosphate phases are observed. This invariance of the MgO/Mg(OH)_2_ pattern, combined with the high Langmuir monolayer capacity and strong affinity constant derived from the isotherm analysis, indicates that phosphate immobilization proceeds predominantly *via* inner-sphere surface complexation and precipitation of poorly crystalline Mg–phosphate species at the Mg–O/Mg–OH interfaces, rather than through bulk transformation of MgO into a well-crystalline phosphate phase.^24^ The regeneration experiments further show that the phosphate removal efficiency of MgO-BC decreases only modestly from ∼99–100% in the first cycle to ∼81% after five adsorption–desorption cycles (Fig. 12b), demonstrating good retention of reactive Mg-rich surface domains and corroborating the high thermal and structural stability inferred from TGA. The XRD observations structurally validate the adsorption behavior deduced from the Langmuir-type isotherms and non-linear PSO kinetics: phosphate removal by MgO-BC is driven by localized high-affinity chemisorption and surface precipitation, rather than non-specific outer-sphere electrostatic interaction.

**Fig. 12:**
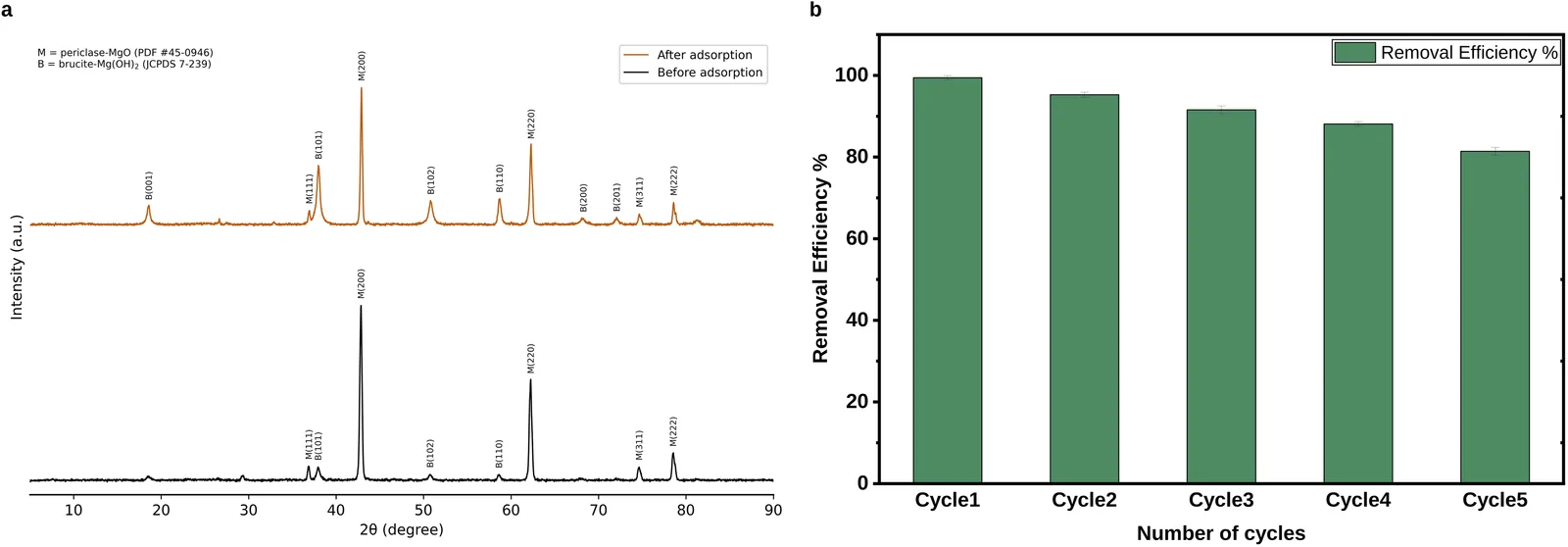
(a) Powder X-ray diffraction (XRD) patterns of MgO-BC before and after phosphate adsorption, (b) reusability of MgO-BC over five successive adsorption–desorption cycles under optimized conditions. Fixed conditions (XRD): Cu Kα radiation (*λ* = 1.5406 Å); 2*θ* range 5–90°; step size 0.02°; scan rate 2° min^−1^. Fixed conditions (reusability): MgO-BC dosage 0.059 g in 10 mL phosphate solution; initial concentration 64.6 mg P per L; pH 8.0; temperature 25 °C; adsorption time 21 h per cycle; desorption with 0.1 mol per L NaOH (10 mL) for 2 h.

The absence of newly resolved crystalline magnesium-phosphate reflections does not exclude the formation of a phosphate-bearing surface deposit because the deposited species may be poorly crystalline or amorphous and below the detection limit of powder XRD. This interpretation is supported by post-adsorption XPS (Fig. 7), which detects surface P 2p and shows changes in the Mg 2p and O 1s chemical environments following phosphate uptake.

### 3.6. Reusability and phosphate recovery

Under the optimized operation conditions, the reusability of the MgO-BC was evaluated for five successive adsorption–desorption cycles, presented in Fig. 12b. Following the initial cycle, the removal efficiency of phosphate steadily declined to 95.26%, 91.56%, 88.12% and 81.41% in cycles 2 through 5, respectively. MgO-BC maintained a removal efficiency more than 80% after five cycles. Desorption capacities, calculated from the phosphate recovered in the alkaline regenerant, ranged between 0.46 and 0.95 mg P per g per cycle. This indicates that a considerable part of the sorbed phosphate can be released and recovered, rather than being permanently immobilized. This cyclical stability aligns with trends reported biochar modified with MgO by direct co-pyrolysis of MgO with the lignocellulosic feedstocks, which typically retains 80–90% of their initial capacity over multiple cycles of alkaline regeneration runs. This sturdy performance is conventionally attributed to the stability of MgO/Mg(OH)_2_ domains and partly reversible nature of surface precipitates of Mg-phosphate. Collectively, the high initial removal efficiency, coupled with the moderate capacity loss with reuse, and the substantial recoverable phosphate, suggests that MgO-BC is an effective, regenerable sorbent, consistent with a surface complexation and surface precipitation mechanism at Mg rich sites, rather than bulk phase irreversible conversion of the biochar matrix.^86–88^

## 4. Conclusion

MgO-modified wattle bark biochar (MgO-BC), synthesized through MgCl_2_ impregnation and co-pyrolysis of *Acacia mearnsii* bark at 600 °C, achieved up to approximately 99.5% phosphate removal under CCD/RSM-optimized conditions and outperformed the unmodified and physically pre-treated biochar variants. The results demonstrate that Mg-containing surface chemistry, rather than surface area alone, governs phosphate uptake in this system. Non-linear Langmuir and pseudo-second-order model fits, together with pH_PZC_, ionic-strength, XRD, and post-adsorption XPS evidence, support a mechanism involving predominantly Mg-centered inner-sphere complexation at Mg–O/Mg–OH sites and poorly crystalline or amorphous surface precipitation. The post-adsorption XPS survey detected surface P 2p at 0.4 at% and showed modified O 1s and Mg 2p envelopes, confirming phosphate interaction with Mg-containing surface sites. MgO-BC retained more than 80% of its initial removal efficiency after five adsorption–desorption cycles, demonstrating regeneration potential. These results position MgO-BC as a low-cost, circular-economy-compatible sorbent for phosphate polishing and recovery.

## Author contributions

Elham Jalali: writing – original draft, visualization, validation, software, resources, project administration, methodology, investigation, funding acquisition, formal analysis, data curation, conceptualization. Elizabeth Erasmus: writing – review & editing, visualization, validation, supervision, software, resources, project administration, methodology, investigation, formal analysis, data curation, conceptualization. Shahab Maghsoudi: writing – review & editing, methodology, investigation. Marietjie Schutte-Smith: writing – review & editing, validation, investigation, and Hendrik. G. Visser: writing – review & editing, visualization, validation, supervision, resources, project administration, methodology, investigation, formal analysis, data curation, conceptualization.

## Conflicts of interest

The authors declare that they have no known competing financial interests or personal relationships that could have appeared to influence the work reported in this paper.

## Supplementary Material

RA-016-D6RA05212F-s001

## Data Availability

The data supporting this article, including the central composite design matrix, the measured phosphate removal responses and the ANOVA results, are provided in the main manuscript. Supplementary information (SI): Fig. S1, which presents Cook's distance and the externally studentized residuals *versus* run number for the quadratic CCD model of phosphate removal. See DOI: https://doi.org/10.1039/d6ra05212f.
