# Supplementary material for: High-affinity phosphate capture on MgO/Mg(OH)2-decorated wattle bark biochar: adsorption mechanism and regeneration performance
Source: RSC Adv. 2026 Oct 12;16(47):62351–70. doi: 10.1039/d6ra05212f (PMC13656217; doi:10.1039/d6ra05212f)
Supplement: Supplementary file 1 [file RA-016-D6RA05212F-s001.pdf]

Supplementary Information (SI) for RSC Advances.
This journal is © The Royal Society of Chemistry

Supplementary Information

High-affinity phosphate capture on $MgO/Mg(OH)_2$-decorated wattle bark biochar: adsorption mechanism and regeneration performance

Elham Jalali[1,2], Elizabeth Erasmus[1], Shahab Maghsoudi[2], Marietjie Schutte-Smith[1], and Hendrik. G. Visser[1]*

1 Chemistry Department, University of the Free State, Bloemfontein, 9300 South Africa

2 Department of Chemistry, Faculty of Science, Shahid Bahonar University of Kerman, Kerman, Iran

Corresponding author: * visserhg@ufs.ac.za (H.G. Visser email)

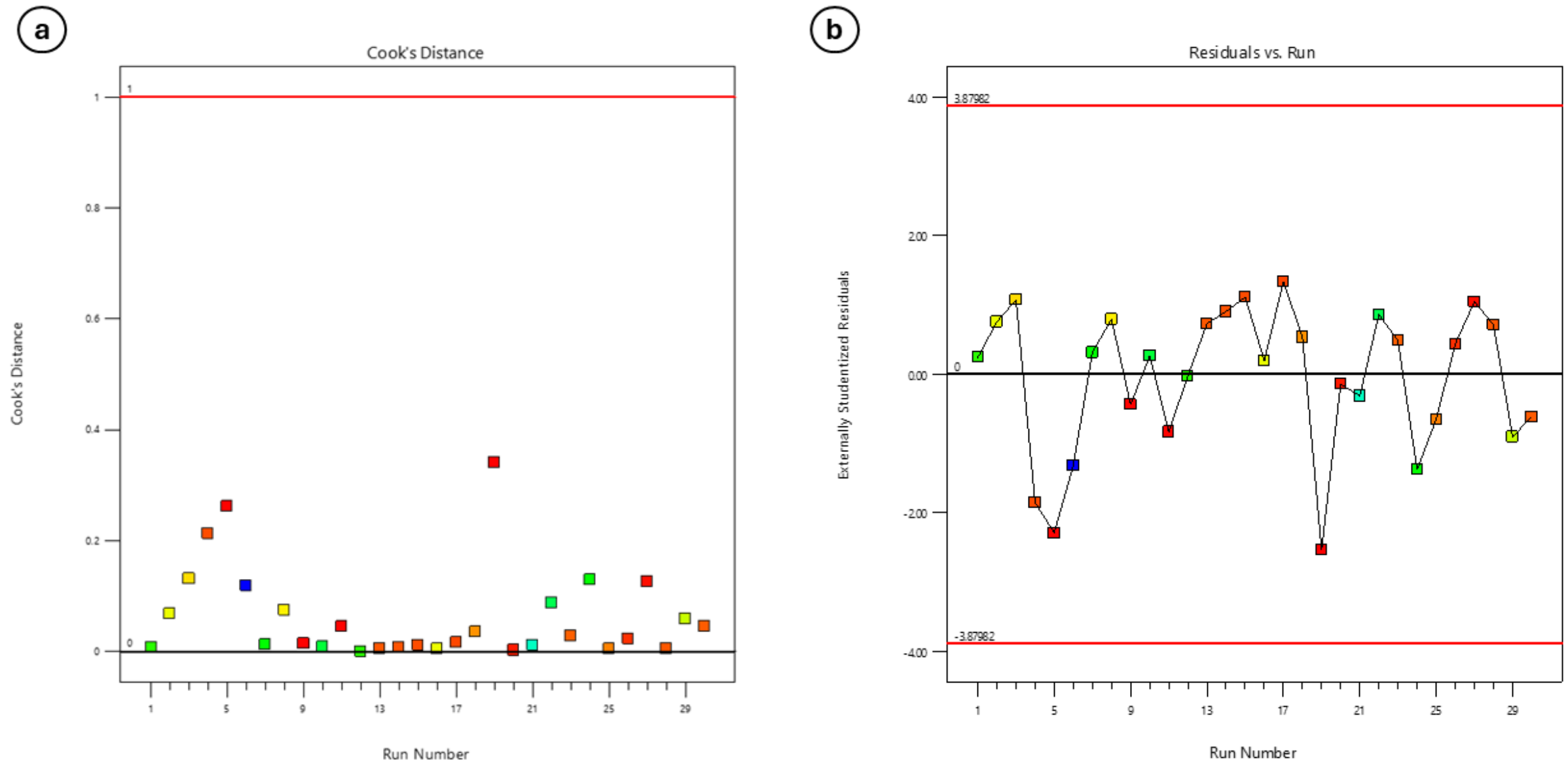

Fig. S1. (a) Externally studentized residuals versus run number for the quadratic CCD model for phosphate removal (R%); all residuals fall within the ±4 limits. (b) Cook's distance versus run number for the same model; all Cook's distances are well below 1, indicating the absence of influential observations.
